# The deLIVERed promises of gene therapy: Past, present, and future of liver-directed gene therapy

**DOI:** 10.1016/j.ymthe.2025.03.041

**Published:** 2025-03-27

**Authors:** Francesco Puzzo, Mark A. Kay

**Affiliations:** 1 Department of Pediatrics, Stanford University, Stanford, CA 94305, USA; 2 Department of Genetics, Stanford University, Stanford, CA 94305, USA

**Keywords:** liver, gene therapy, viral vectors, AAV, clinical trials

## Abstract

Gene therapy has revolutionized modern medicine by offering innovative treatments for genetic and acquired diseases. The liver has been and continues as a prime target for *in vivo* gene therapy due to its essential biological functions, vascular access to the major target cell (hepatocytes), and relatively immunotolerant environment. Adeno-associated virus (AAV) vectors have become the cornerstone of liver-directed therapies, demonstrating remarkable success in conditions such as hemophilia A and B, with US Food and Drug Administration (FDA)-approved therapies like etranacogene dezaparvovec, Beqvez, and Roctavian marking milestones in the field. Despite these advances, challenges persist, including vector immunogenicity, species-specific barriers, and high manufacturing costs. Innovative strategies, such as capsid engineering, immune modulation, and novel delivery systems, are continuing to address these issues in expanding the scope of therapeutic applications. Some of the challenges with many new therapies result in the discordance between preclinical success and translation into humans. The advent of various genome-editing tools to repair genomic mutations or insert therapeutic DNAs into precise locations in the genome further enhances the potential for a single-dose medicine that will offer durable life-long therapeutic treatments. As advancements accelerate, liver-targeted gene therapy is poised to continue to transform the treatment landscape for both genetic and acquired disorders, for which unmet challenges remain.

## Graphical abstract

**Figure:**
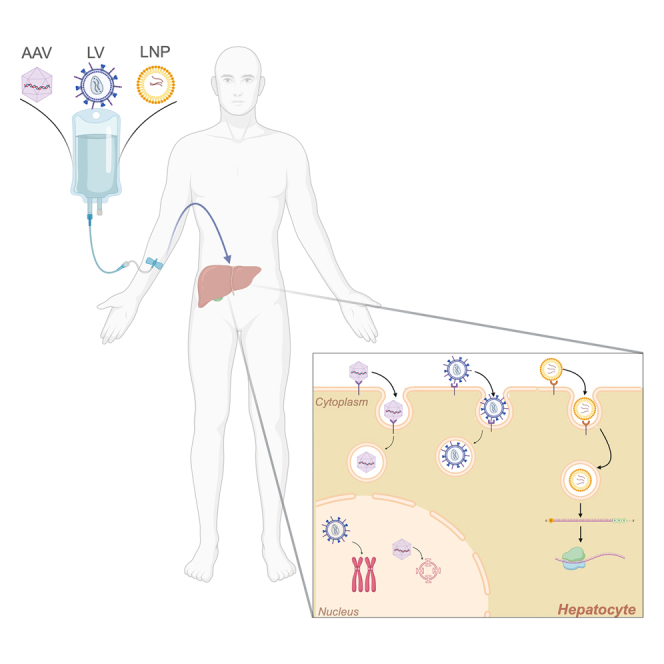

## Teaser

Puzzo and Kay review liver-based gene therapeutics from the early days to present times.

## Introduction

Gene therapy offers the potential to treat and potentially cure a wide array of genetic and acquired disorders.^1^ The earliest form of liver gene therapy involved liver or hepatocellular transplantation for individuals with severe untreatable genetic disorders. Here, we restrict our review to the autologous introduction of nucleic acids into hepatocytes with the goal of modifying genes to cure diseases by either replacing or fixing defective genes, inactivating dysfunctional ones, or introducing new genes for the treatment of both genetic and acquired diseases. Historically, the genetic transfer “vehicles” consisted of either plasmid DNA (non-viral) or viral-based systems. Gene transfer products can be delivered either *ex vivo*, by modifying cells outside the body before reinfusion, or *in vivo*, by directly modifying cells within the body.^2^

**Figure 1:**
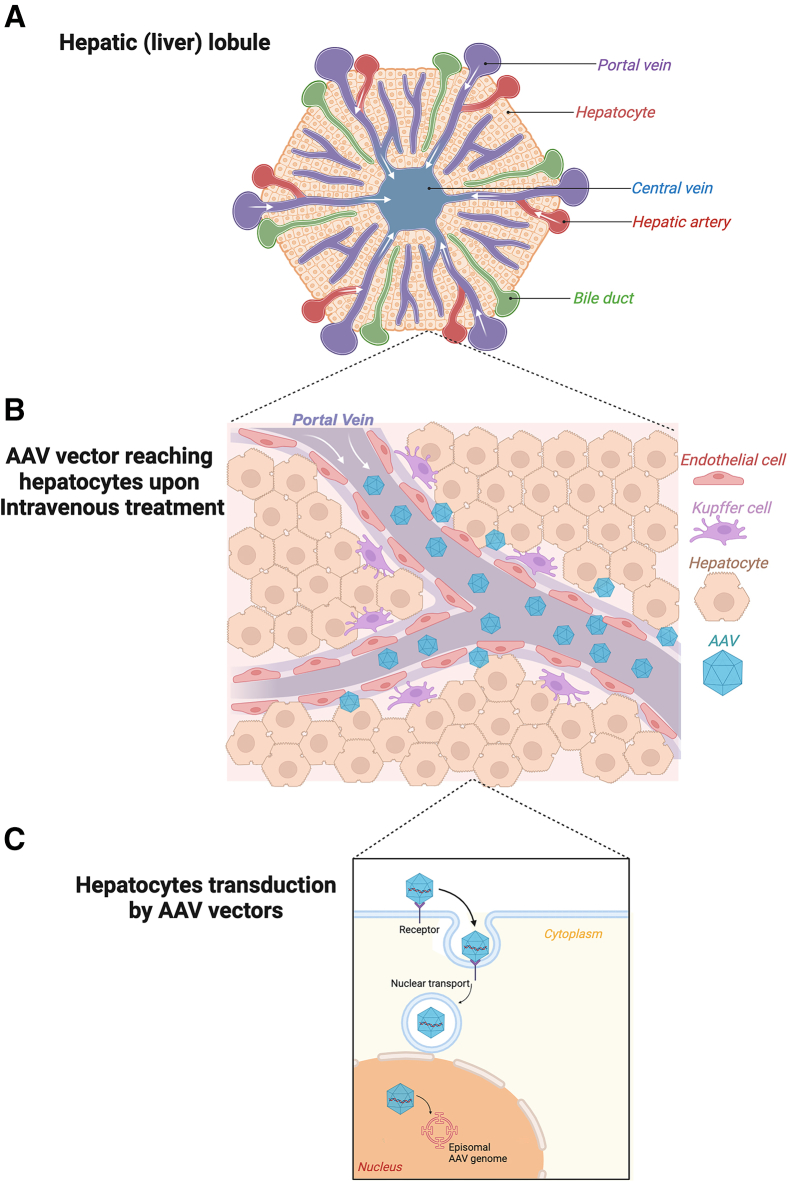
AAV-mediated transduction of hepatocytes upon systemic vector administration (A) The hepatic lobule, the fundamental functional unit of the liver, is illustrated as a hexagonal structure. It features a central vein at its core and portal triads situated at its peripheral vertices. Blood flows from the portal vein and hepatic artery, both components of the portal triad, through the sinusoids toward the central vein. (B) Upon intravenous administration, adeno-associated virus (AAV) vectors circulate through the portal vein and enter the sinusoidal spaces of the liver. Within these spaces, the vectors interact with sinusoidal endothelial cells and Kupffer cells before successfully transducing hepatocytes. (C) The mechanism of hepatocyte transduction by AAV vectors is depicted. The vectors bind to specific cell surface receptors on hepatocytes, initiating receptor-mediated endocytosis. Following internalization, the AAV particles undergo intracellular trafficking and are subsequently transported to the nucleus. Within the nucleus, the AAV genomes persist as episomal DNA, facilitating sustained expression of the delivered transgene.

The liver plays a central role in numerous crucial biological processes in regulating many metabolic pathways including carbohydrates, amino acids, and lipids. It also produces many secreted proteins including those required for hemostasis. Moreover, the intrinsic tolerogenic properties of hepatocytes promote immune tolerance toward the transgene expressed within these cells, making the liver an attractive target for gene therapy. The liver also plays a major role in detoxification and removal of unneeded circulating compounds. This requires a vasculature structure with open fenestrations allowing hepatocytes to come in contact with large macromolecular structures such as gene transfer vectors^3^ (Figure 1). Taken together, the liver has become a primary target for gene-therapy applications. Thus, liver-directed gene therapy holds promises for once-in-a-lifetime treatment for numerous inherited metabolic disorders (i.e., Fabry disease, mucopolysaccharidoses [MPS], Gaucher disease, Crigler-Najjar syndrome), coagulation factor deficiencies (hemophilia A and B), and other conditions such as cancer or viral infections.^4^

Adeno-associated virus (AAV) vectors have emerged as the leading delivery system for liver-directed gene therapy due to their hepatotropic nature, efficiency in transducing hepatocytes, relatively low immunogenicity, and ability to mediate long-term transgene expression.^5^ Recent years have witnessed significant progress in the field, with several liver-targeted gene therapies advancing through clinical trials. Notably, treatments for hemophilia A and B have shown exceptional results that led to their approval. Indeed, there are currently three different approved gene-therapy products for the treatment of hemophilia, etranacogene dezaparvovec and Beqvez for hemophilia B, and Roctavian for hemophilia A, marking a milestone in the field of AAV-mediated liver gene therapy.^6^ However, despite these advancements, several challenges remain to broaden the transition from bench to bedside. These involve managing immune responses to the vector and transgene product,^7^ ensuring long-term efficacy,^8^^,^^9^ addressing potential genotoxicity concerns,^10^ improving the efficacy of hepatocytes transduction,^11^ and finding an effective strategy to re-dose patients with therapeutic AAV vectors if needed.^12^ The occurrence of severe adverse events in some high-dose AAV trials has highlighted the need for careful evaluation of safety profiles, particularly in patients with pre-existing liver conditions.^13^^,^^14^ As the field progresses, researchers are exploring innovative approaches to overcome these hurdles, including the development of more efficient and specific viral vectors, gene-editing technologies, and strategies to enable vector re-administration.

In this review, we provide a historical overview on the field of liver gene therapy and the multitude of viral and non-viral approaches that are currently being investigated in preclinical settings. Then we highlight the real-life data emerged from the several clinical trials conducted in the last decade. Finally, we will discuss the perspectives of the AAV-mediated liver gene therapy and how the new technologies might shape the path toward better, safer, and more effective therapies.

### History of liver gene therapy: Failures and accomplishments

The inception of the idea for gene therapy started to gain traction in the 1980s following the routine development of techniques for cloning eukaryotic expression cassettes. The primary goal was to introduce an expression cassette (e.g., gene addition) to restore the function resulting from a missing protein, generally due to an inherited recessive monogenic disorder. Most of the effort in the early days was focused on gene transfer either into hematopoietic derived progenitor/stem cells or liver. In the mid to late 1980s, researchers developed retroviral vectors packaged in an amphotropic envelope that were capable of transferring expression cassettes into mammalian cells,^15^ and this propelled many gene-therapy attempts. This advancement represented an important step forward for liver-targeted approaches, as this organ is implicated in numerous inborn errors of metabolism, making it a critical target for intervention.

Early on, there were two general approaches being considered for liver-directed gene therapy: *ex vivo* vs. *in vivo* delivery. The *in vivo* approach, which constitutes the primary focus of this review, involves the direct administration of a vector into a living organism to facilitate gene transfer and expression specifically in hepatocytes. In contrast, the *ex vivo* strategy involves removing a lobe of the liver, isolating hepatocytes on culture dishes, genetically modifying them using a retroviral vector, and re-implanting the cells into the liver via splenic transplant or vascular delivery routes. Preclinical studies utilizing large animal models, such as dogs for liver-derived secreted proteins and Watanabe rabbits for low-density lipoprotein receptor (LDLR)-related applications, were actively pursued.^16^^,^^17^ A small clinical trial for familial hypercholesterolemia (LDLR deficiency) was performed,^18^ but, due the limited clinical efficacy, enormous complexity, and safety issues, these approaches were abandoned.

#### To integrate or remain episomal

Gene transfer vectors facilitate either episomal maintenance or integration-based incorporation of DNA into the host genome. Unlike the hematopoietic system, it has been established that life-long expression in the liver does not necessitate the genetic modification of hepatic stem cells.^19^ Based on liver regeneration studies, modifications to the genome in a mature hepatocyte will result in life-long expression provided that the genetic modification is not detrimental to cell survival. In fact, there are several liver genetic disorders for which replacement or correction of the defective gene leads to a selective advantage and liver repopulation with the genetically modified cells. The most compelling example is tyrosinemia type 1, where genetic modification of as few as 1,000 cells has been shown to result in successful repopulation of the mouse model for this condition.^20^ This model was extensively studied when *in vivo* vector integration studies were still inefficient for most liver hepatodeficiency disorders^21^^,^^22^ Nonetheless, in quiescent hepatocytes, episomal gene transfer may provide long-term and perhaps life-long transgene expression.

### The vectors for *in vivo* delivery

#### Murine-based retroviruses (integrating vectors)

Given that Moloney based retroviral transduction required cellular proliferation, direct *in vivo* retroviral administration through the liver vasculature approaches were assessed in both rodents^23^ and hemophilia B dogs^24^ after a two-thirds surgical partial hepatectomy. This therapeutic strategy successfully achieved stable expression of canine factor IX in hemophilia B dogs,^25^ but the relatively low levels of circulating factor IX, combined with the complexity and safety concerns associated with the approach, ultimately led to its discontinuation. Nevertheless, a phase I intravenous dose escalation trial using a retrovirus expressing a B-domain-deleted factor VIII protein was assessed in hemophilia A patients but was unsuccessful.^26^

#### Lentiviruses (integrating vectors)

**Figure 2:**
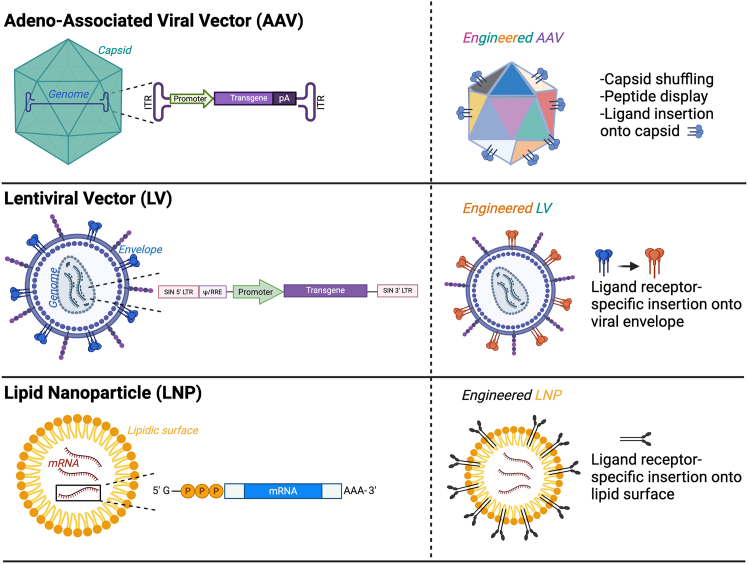
Engineering strategies to enhance the efficacy and safety of gene-therapy vectors (Top) On the left, the native AAV vector without modification. On the right, engineered AAVs are depicted with enhanced features such as capsid shuffling, peptide display, and ligand insertion onto the capsid surface. These modifications enable improved cell-specific targeting and delivery efficiency. (Middle) The native lentiviral vector is shown on the left. On the right, engineered lentiviral vectors are shown with ligand receptor-specific modifications on the viral envelope. These modifications improve the targeting of specific cell types. (Bottom) The left panel depicts a standard lipid nanoparticle, comprising a lipidic surface encapsulating therapeutic mRNA. On the right, engineered LNPs are shown with ligand receptor-specific insertions on the lipid surface, allowing for selective targeting of specific cells and tissues.

A renewed interest in the use of retroviral vectors emerged with the development of lentiviral (LV) vectors.^27^ Their use was attempted early on for *in vivo* liver gene transfer because of their ability to transduce quiescent cells.^28^ However, the early-generation vectors, while able to transduce quiescent cells, had more robust transduction of cycling hepatocytes.^29^ Nevertheless, improvements made in the vector over the years have resulted in their wide use in non-liver *ex vivo* gene-therapy approaches. Another advantage is the low prevalence of neutralizing antibodies in the population and their high packaging capacity, allowing for the incorporation of expression cassettes, up to 9 kb.^30^ Their ability to integrate stably into the host genome enables sustained expression of therapeutic genes, making them suitable for targeting proliferative and developing tissues, such as the liver in pediatric patients.^31^ However, despite their different integrative pattern compared to Moloney retroviruses, they still carry a potential risk of insertional mutagenesis, as observed in certain non-liver clinical trials.^32^ Additionally, the structural components of the envelope commonly used based on vesicular stomatitis virus G glycoprotein (VSV-G) can elicit significant inflammatory and immune responses following *in vivo* administration.^33^^,^^34^ By inserting immunomodulatory proteins into the envelope, a reduction in some of these responses have been observed renewing their interest in use for liver-directed gene therapies^35^ (Figure 2). One study demonstrated that engineering producer cell lines to prevent the incorporation of major histocompatibility complex class I (MHC-I) molecules onto LVs can reduce immunogenicity *in vivo*.^36^ Another approach involved the addition of CD47, a phagocytosis-inhibiting molecule, to the LV surface, which was shown to enhance *in vivo* liver gene transfer in NHPs.^37^ Thus, although LVs have not yet been evaluated in an *in vivo* clinical context, preclinical studies in animal models for hemophilia A and B provide evidence supporting the safety and efficacy of these approaches for future liver-directed *in vivo* clinical trials.^38^^,^^39^

#### Adenoviruses (episomal vectors)

First-generation recombinant adenoviral vectors, characterized by E1 and/or E1/E3 gene deletions and episomal-based expression, were developed in the same time frame retroviruses were tested for *in vivo* gene transfer. These vectors were first used to successfully correct the urea cycle defect associated with ornithine transcarbamylase (OTC) deficiency in a mouse model.^40^ Recombinant adenoviral vectors became highly studied and were used in a canine model of hemophilia B to provide high, albeit transient, levels of canine factor IX.^41^ Toxicity in animals became apparent and there were major efforts to remove portions or all the adenoviral coding sequences in the vectors.^42^^,^^43^ This was because there were concerns that leaky expression from the remaining adenoviral genes induced toxicity, vector DNA replication, and T cell-dependent immune responses. With the tragic death of Jessie Gelsinger,^44^ an 18-year-old with a mild form of ornithine transcarbamylase deficiency, treated with an early-generation recombinant adenoviral vector, the enthusiasm for the use of these vectors for *in vivo* hepatic gene therapy was greatly diminished. However, continued work to remove all the adenoviral genes not only reduced toxicity but expanded the DNA cargo size to nearly 35 kb. Moreover, the helper-dependent gene-deleted adenoviral vectors were successfully delivered in large animal models by intrahepatic administration using balloon catheters.^45^ However, these vectors were not easy to manufacture and, while they showed improved toxicity profiles, they were not eliminated.^46^ Due to the issues mentioned and the increased interest in AAV vectors, recombinant adenoviral vectors were not rigorously pursued for liver-directed gene replacement.

Several other viral vectors, including those derived from herpes simplex virus^47^ and SV-40,^48^ were used for liver gene-transfer approaches but without much success due to lack of efficacy in preclinical studies.

#### Non-viral vectors (episomal and integrating vectors)

During the same time frame, there was a great deal of effort toward developing non-viral vectors for liver-directed gene therapy. These primarily consisted of canonical plasmid DNAs (episomal) and then DNA transposable-element-based vectors (integrating). Delivery was and arguably still is the major limitation to non-viral-based DNA delivery into the liver. In the 1990s, there were encouraging results using DNA-protein conjugates primarily to the asialoglycoprotein, which is taken up exclusively by the liver.^49^ However, over the following years, none of these studies were reproducible in other labs. Importantly, the Liu^50^ and Wolff^51^ laboratories independently published a technique called hydrodynamic transfection where a relatively large volume of a DNA solution in an aqueous buffer was injected by high-pressure tail-vein infusion resulting in DNA transfection in up to 20%–30% of mouse hepatocytes. This resulted in two general lines of experimentation. The first aimed to develop this technique in large animals as a precursor to clinical development. This turned out to be unsuccessful even though there was a limited attempt to treat patients.^52^ In the second, this technique provided a means to study various expression cassettes in a primary tissue and further established the discrepancies in promoter expression in an intact liver *in vivo* and cultured primary hepatocytes. Moreover, the same expression cassettes in a recombinant AAV vector vs. a DNA plasmid provided very different expression profiles. The AAV-mediated gene transfer resulted in long-term expression, while the plasmid-mediated expression was short lived and subsequently shown to be a result of transcriptional shut-off from the plasmid. This was demonstrated to be due to the bacterial backbone DNA and resulted in the development of minicircle DNA vectors, which were plasmids devoid of the bacterial backbone and provided persistent expression.^53^^,^^54^ In addition, DNA transposon vectors for integration of therapeutic genes had also been used in preclinical studies.^55^ Nevertheless, the major limitation to non-viral DNA expression vectors remains a clinically relevant means to achieve nuclear delivery of the DNA cargo.

#### AAVs (episomal vectors)

While AAVs were discovered in 1965,^56^ their first use for transgene transfer was demonstrated in 1984.^57^ In the early 1990s, there was new excitement for the development of AAV vectors based on the idea that they could be engineered to integrate into a safe spot in the human genome.^58^ However, integration required the co-expression of the AAV-Rep protein, which led to toxicity and yet still resulted in very low levels of integration. Over the pursuing years, the demonstration that AAV vectors were able to deliver therapeutic and potentially curative levels of factor IX in mice and dogs without any toxicity^59^^,^^60^^,^^61^ was a major breakthrough for the gene-therapy field. In addition, it became clear that the major stable vector DNA formed in transduced cells was double-stranded episomal^19^ with a small percentage of genomes integrated into the host genome.^10^

Over almost three decades of research led to the isolation of novel serotypes from various sources including NHPs,^62^ interspecies AAV capsid molecular shuffling,^63^ and ligand conjugation to capsids^64^^,^^65^ by academic and commercial groups in order to develop AAV vectors with improved properties.^5^^,^^66^

#### Virus-like particles (episomal vectors)

Virus-like particles (VLPs) are recently emerging as promising delivery systems for nucleic acids and proteins.^67^ VLPs are composed of non-infectious viral particles, with a structure largely based on retroviruses. Preclinical models have shown encouraging results in the *in vivo* delivery of mRNA or proteins,^68^ and efforts are ongoing to further enhance VLP efficacy and optimize their use.^69^ Nevertheless, unlike lipid nanoparticles (LNPs), VLPs possess a protein core on their surface, which may limit their potential for re-administration due to possible immune responses.

### Ongoing approaches for *in vivo* liver gene therapy

Nowadays, the vectors employed for the delivery of genetic material in preclinical research for liver-targeted gene transfer primarily consist of AAVs, LV vectors, and non-viral systems. Each approach has unique properties and, in some cases, use combinations of these systems to enhance their therapeutic efficacy and expand their use to a variety of medical indications.

#### AAV vectors

AAV vectors catalyzed a resurgence in gene therapy, following a period of setbacks that hampered progress in the field.^44^^,^^70^ Due to their non-pathogenic nature and inherent liver uptake and tropism, AAV vectors are currently the preferred vehicle to deliver therapeutic genes for a broad spectrum of diseases.^4^ These include not only genetic disorders^71^ but also viral infection^72^ and secretion of antibodies for passive immunity strategies.^73^^,^^74^ Furthermore, numerous clinical trials have shown both the safety and long-term efficacy of AAV administration in patients.^4^ Nonetheless, there are some intrinsic limitations for the translation of the AAV to the clinic that researchers are still trying to address at preclinical stages.

#### Preclinical models

The majority of preclinical studies are conducted in small rodents, primarily mice and rats. However, natural AAV serotypes that effectively transduce murine cells do not exhibit equivalent efficiency in transducing humans and non-human primate (NHP) cells.^75^^,^^76^ As a result, alternative preclinical models in an attempt to better predict transduction in humans have been studied, including a chimeric humanized mouse liver^77^ and even whole human liver explants.^78^^,^^79^ While no model is perfect and they all have their limitations, these two tend to more accurately predict human transduction efficacies.

In 2023, a committee of experts published a report providing recommendations on the adoption of novel alternative methods (NAMs) to advance biomedical research. These recommendations were subsequently endorsed by the Advisory Committee to the Director and formally approved by the NIH in February 2024.^80^ Although the transition from animal models to alternative methods is anticipated to be a gradual but ultimately necessary process, significant progress has already been achieved in this area.

The development and application of three-dimensional (3D) cell cultures, commonly referred to as organoids, have introduced a promising platform for preclinical evaluation of gene-therapy products.^81^ Liver organoids can be derived from primary liver tissue or from pluripotent stem cells, which are in turn generated from accessible sources such as skin biopsies, blood cells, or renal epithelial cells found in urine.^82^^,^^83^ Furthermore, advancements in organ-on-a-chip technology have enabled the integration of additional cellular complexity into liver organoids by incorporating other liver-resident cell types, including endothelial cells and Kupffer cells.^84^^,^^85^

A landmark study published in 2022 demonstrated the successful transplantation of a human liver graft that had been preserved *ex vivo* for several days.^86^ Thus, the same *ex vivo* organ maintenance protocol has recently been utilized to evaluate AAV vectors in intact human liver tissue.^79^

These platforms provide the ability to assess vector biodistribution across distinct liver cell populations, such as hepatocytes, endothelial cells, and Kupffer cells. Additionally, they facilitate the engineering and optimization of more potent cell-specific vectors while enabling evaluation of potential inflammatory and immune responses. Liver cells derived from individuals with hepatic diseases can also serve as a model to assess the efficacy of gene therapies.^78^ Together, organoids and *ex vivo* liver platforms hold the potential to overcome species-specific barriers in the preclinical evaluation of liver-targeted gene therapies while significantly reducing the reliance on animal models.

There has been a growing interest in engineering AAV capsids through methods such as DNA shuffling and peptide display,^5^ aiming to enhance hepatic transduction efficiency in both NHP and human hepatocytes, thus facilitating clinical translation. Interestingly, some of the newly engineered AAV vectors display preferential tropism toward primate hepatocytes (both human and NHP), while showing minimal transduction in murine liver cells.^75^^,^^87^ This phenomenon has been hypothesized to result from species-specific intracellular processing mechanisms^88^ or epigenetic regulation of the AAV genome unique to each species.^76^ To facilitate the clinical translation of AAVs, a proposed strategy involves the selection of AAV capsids across multiple species, including mice, NHPs, and primary human cells.^89^ This approach aims to identify capsids capable of efficiently transducing target organs in all tested species, thereby enabling its application in both preclinical studies (mice and NHPs) and clinical settings (humans). The ongoing research into these species-specific mechanisms, along with further vector engineering to optimize efficacy across both preclinical and clinical models (Figure 2), will certainly advance and facilitate the development of safer and more effective AAV-based liver gene therapies.

#### Pre-existing immunity

An important limitation of AAV vectors is their immunogenicity. Although natural AAV infections in humans are typically asymptomatic,^90^ they elicit an immune response that leads to the generation of neutralizing antibodies (NAbs) against the AAV capsid.^91^ The presence of these NAbs inhibits the transduction of target tissues upon vector administration, making the gene-therapy product ineffective.^92^ Consequently, all current AAV-based clinical trials exclude individuals with high NAb titers.^93^ Additionally, systemic administration of an initial AAV dose induces NAb production, which effectively precludes the possibility of subsequent AAV treatments.^94^ Nevertheless, researchers are actively exploring various strategies to address this challenge, encompassing physical approaches (e.g., plasmapheresis), enzymatic methods (e.g., IdeS), and immunosuppressive interventions (e.g., ImmTOR).

Plasmapheresis is a medical procedure designed to separate plasma from other blood components. Hemapheresis represents a more advanced approach, enabling the isolation and collection of specific blood constituents. Both methods have been extensively studied in the context of *in vivo* gene therapy, specifically for their role in depleting the immunoglobulin fraction of blood, which contains antibodies.^95^^,^^96^ In patients with high levels of NAbs, this depletion creates a window during which AAV vectors can be administered without interference from pre-existing anti-AAV antibodies. However, this process is not without substantial medical risks, which increase with the number of plasmapheresis cycles required, including immune suppression, which in turn is associated with an increased risk of infection.^97^^,^^98^

Recently, a protease known as IdeS, which cleaves human immunoglobulin G (IgG) into F(ab')2 and Fc fragments, has been identified in the bacterium *Streptococcus pyogenes*.^99^ This enzyme is currently under clinical investigation as a therapeutic agent for autoimmune diseases and organ transplantation.^100^^,^^101^ In preclinical models, including murine and NHPs studies, IdeS administration has demonstrated potent depletion of circulating NAbs, thereby enabling effective administration and transduction of AAV vectors in the context of both pre-existing NAbs and vector re-administration.^102^^,^^103^ Ongoing clinical trials in healthy subjects have reported minor adverse events and provided insights into the pharmacokinetics of this strategy, demonstrating approximately a 90% reduction in IgG levels within 6 h post treatment. These studies have also identified a 1-week window during which sustained low levels of circulating IgG could possibly make effective the administration of AAV vectors.^104^^,^^105^

A recent approach to address the challenge of NAbs has been the development of immunomodulatory nanoparticles which induce an antigen-mediated proliferation of regulatory T cells (Tregs), such as ImmTOR.^106^ Preclinical studies have demonstrated that ImmTOR enables AAV vector administration in animal models that developed high titers of NAbs.^107^ Furthermore, ImmTOR enabled a second vector administration following an initial treatment with AAVs.^108^ Finally, another immune-suppressive strategy based on the use of an antibody blocking the neonatal Fc receptor has demonstrated encouraging results in re-administrating AAV vector in seropositive animal models.^109^

Overall, the continued refinement and optimization of these strategies hold strong promises for broadening the use of AAV vectors in individuals with high NAb titers who are currently excluded from clinical trials. Additionally, these advancements are expected to support the potential for re-administration of AAV vectors, expanding the clinical applications of AAV-based therapies.

#### Integration issues

While most of the stable AAV genomes are episomal, it became clear a small percentage of the AAV genomes can integrate into the host cell genome. Crude early estimates determined by inducing liver regeneration in AAV treated animals suggested that as many as 5% of the stable vector genomes were integrated,^19^ and limited sequencing demonstrated integration occurred in transcriptionally active sites.^110^ David Russell and Mark Sands,^111^ and then Chandler and Venditti,^112^ showed that neonatal mice injected with AAV vectors containing a strong liver promoter developed high rates of hepatocellular carcinoma in most cases due to activation of the *Rian* locus in the liver. The *Rian* locus is imprinted, developmentally regulated, and encodes oncogenic non-coding RNAs. The Grompe lab used the chimeric humanized mouse liver model to show integration rates of 1%–3% in human hepatocytes.^113^ Despite multiple recent workshops and studies, there remains a great deal of debate about the integration frequency and oncogenesis risk with AAV administration in human liver. In general, the consensus is that the risk in humans is lower than what has been observed in rodents.^10^ Moreover, it is important to keep in mind that a human liver contains roughly 2 × 10^11^ hepatocytes and, even at an integration rate of 1.0%, represents 10 billion integration events.^114^

#### Vector persistence

Multiple studies have demonstrated that liver-directed gene transfer of AAVs in adult animals resulted in long-term transgene expression. In contrast, progressive loss of the AAV genome has been observed in neonatal murine and NHP animals attributed to organ growth and cellular division.^115^^,^^116^ Consequently, the episomal nature of the AAV genome has thus far posed a challenge for administering these therapeutic vectors to pediatric patients. Nonetheless, the aforementioned efforts to develop strategies for vector re-administration following initial AAV treatment are expected to result in effective therapeutic dosing in pediatric subjects, with the potential for supplementary treatment in adulthood once hepatic growth has stabilized.

### Genome-editing approaches

Genome editing encompasses many entities. They include methods to achieve gene inactivation by disruption of the coding sequence or inactivation of gene expression, turning up the expression of a gene, fixing a specific genetic mutation, and insertion of DNA sequences (e.g., coding regions or expression cassettes) into specific regions of the genome. During the early decades of the 21st century, zinc-finger nucleases (ZFNs) and transcription activator-like effector nucleases (TALENs) were employed to cut specific sequences in the genome and dominated the gene-editing field.^117^ However, the discovery of the CRISPR-Cas9 system, with its exceptional versatility, significantly shifted the paradigm of genome editing.^118^ CRISPR/sCas9 has emerged as the primary nuclease employed in therapeutic genome-editing strategies and in hematopoietic derived cells. Casgevy (exagamglogene autotemcel) has recently been approved for the treatment of sickle cell disease and β--thalassemia.^119^^,^^120^^,^^121^

The earlier strategies employed the use of recombinant AAV vectors to express the nuclease and, in the case of CRISPR-Cas9, the nuclease and guide RNAs to induce either single-strand breaks (SSBs) or double-strand breaks (DSBs) in the host genome, combined with single-stranded AAV (ssAAV) vectors serving as DNA donors for genome editing.^122^ However, there were legitimate concerns raised about the continued expression from the AAV- nuclease from an episome or due to the increased risk of the nuclease expressing vector inserting into a nuclease-mediated DNA break.^123^ As RNA delivery into the liver had become more routine in the clinic originally as an RNAi therapeutic,^124^ CRISPR-Cas9 delivered as messenger RNA (mRNA)/guide RNA (gRNA) complex encapsulated in LNPs^125^ or, more recently, pre-assembled CRISPR-Cas9-gRNA ribonucleoprotein complexes^126^ have become the preferred approaches. This is because the transient presence of the editing molecules greatly reduces the risk of detrimental off-targeting events.

LNPs have been extensively studied as delivery vehicles for nucleic acids for several decades. Early formulations, however, were associated with significant hepatotoxicity in preclinical models.^127^ Additionally, a recent study highlighted a critical limitation of LNP-mediated delivery, showing that only a small proportion of the encapsulated mRNA is efficiently released into the cytosol, underscoring the challenge of overcoming endosomal escape.^128^ Despite these pitfalls, research in this field has remained highly active, with continuous efforts aimed at developing safer and more effective LNP systems.^129^ In addition, the remarkable success of SARS-CoV-2 mRNA vaccines has further reinvigorated interest in LNPs as platforms for genetic material delivery.^130^ Recent advancements, including the design of novel ionizable lipids and the incorporation of tissue-specific targeting ligands (e.g., GalNAc), have significantly enhanced the safety profile and liver-specific delivery capabilities of LNPs^105^^,^^131^^,^^132^ (Figure 2). Furthermore, preclinical animal studies have demonstrated that LNP re-administration is safe and does not induce adverse immune-inflammatory responses.^133^^,^^134^ In a recent study conducted in mice and NHPs, it was demonstrated that co-delivery of *Sleeping Beauty* transposase mRNA encapsulated in LNPs, in combination with ssAAV as donor DNA, achieved high levels of genome editing in the liver.^135^ Recently, the development of novel and potentially safer genome-editing tools, such as prime editors and base editors, has generated significant interest in liver-targeted *in vivo* gene editing using LNPs.^136^^,^^137^^,^^138^

Furthermore, a novel strategy leveraging epigenetic chromatin modifications has been developed for gene-editing applications.^139^ This approach enables the delivery of epigenome editors by LNP/mRNA complexes to achieve stable and permanent silencing of gene expression in rodents as well as in larger animal models, including NHPs.^140^ Importantly, clinical trials aimed at achieving the permanent silencing of the hepatitis B virus (HBV) to treat infected individuals are anticipated to start in the second quarter of 2025.^141^ Consequently, the application of LNPs for delivering genome-editing moieties for liver diseases has already advanced to clinical stages, as will be discussed in the following section.

### From the bench to the bedside: Into the clinic

Nowadays, most clinical trials for liver-targeted gene therapies, as well as the three approved therapies—etranacogene dezaparvovec, Beqvez, and Roctavian—rely on the intravenous administration of AAV vectors. Only recently have LNPs been utilized in such therapeutic approaches, with several clinical trials currently in progress. To date, no lentiviral-based *in vivo* gene transfer has advanced to the clinical stage (Table 1).

**Table 1:** Liver gene-therapy clinical trails

Medical indication	Drug	Dose	Clinical phase
Hemophilia B	Hemgenix (ssAAV5-hFIXPadua)	2 × 1013 vg/kg	approved
	Beqvez (ssAAV74-hFIXPadua)	5 × 1011 vg/kg	approved
	FLT180a (ssAAVS3-hFIXPadua)	3 × 1011–1 × 10^12^ vg/kg	phase I/II (terminated) NCT03641703
	REGV131-LNP1265(LNP-CRISPR-CAS9mRNA+ ssAAV8 hFIX)	N/A	phase I/II (ongoing) NCT06379789
Hemophilia A	Roctavian (ssAAV5-BDD/hFVIIIwt)	6 × 10^13^ vg/kg	approved
	dirloctocogene samoparvovec (ssAAVLK03-BDD/hFVIIIwt)	5 × 1011–2 × 10^12^ vg/kg	phase I/II (completed) phase III (ongoing) NCT06379789
	giroctocogene fitelparvovec (ssAAV6-BDD/hFVIIIwt)	3 × 10^13^ vg/kg	phase I/II (completed) phase III (ongoing) NCT03587116
MPS VI	ssAAV8-hARSB	6 × 10^11^–6 × 10^12^ vg/kg	phase I/II (completed) NCT03173521
Acute intermittent porphyria	ssAAV5-hPBGD	5 × 10^11^–1.8 × 10^13^ vg/kg	phase I/II (terminated) NCT02082860
Crigler-Najar	GNT0003 (ssAAV8-hUGT1A1)	2 × 10^12^ and 5 × 10^12^ vg/kg	phase I/II (ongoing) NCT03466463
GSDIa	DTX-401 (ssAAV8-hG6PC)	1 × 10^13^ vg/kg	phase I/II (completed) phase III (ongoing) NCT03970278
Gaucher	FLT201 (ssAAVS3-hGCase85)	4.5 × 10^11^ vg/kg	phase I/II (ongoing) NCT05324943
Fabry	ST-920 (ssAAV6-hGLA)	2.63 × 10^12^ vg/kg	phase I/II (ongoing) NCT04046224
Pompe	ACTUS-101 (ssAAV8-hGAA)	1.6 × 10^12^ vg/kg	phase I/II (terminated) NCT03533673
	SPK-3006	N/A	phase I/II (terminated) NCT04093349
Wilson	UX701 (ssAAV9-hATP7B)	5 × 10^12^–2 × 10^13^ vg/kg	phase I/II (ongoing) NCT04884815
	VTX-801 (AAV-hATP7B minigene)	N/A	phase I/II (ongoing) NCT04537377
Propionic acidemia	mRNA-3927 (LNP-hPCCA/hPCCB mRNA)	0.3–0.9 mg/kg every 2weeks	phase I/II (ongoing) NCT04159103
ATTR amyloidosis	NTLA-2001 (LNP-CRISPR-CAS9+ hTTRgRNA mRNA)	0.1–0.3 mg/kg	phase I/II (completed) phase III (ongoing) NCT06672237
Hereditary angioedema	NTLA-2002 (LNP-CRISPR-CAS9+ hKLKB1gRNA mRNA)	50 mg	phase I/II (completed) phase III (ongoing) NCT06634420
Hypercholesterolemia	VERVE-101 (LNP-Adenine Base Editor + hPCSK9gRNA mRNA)	0.1–0.6 mg/kg	phase I/II (terminated) NCT05398029
	VERVE-102 (LNP/GalNac-Adenine Base Editor + hPCSK9gRNA mRNA)	N/A	phase I/II (ongoing) NCT06164730
Alpha-1 antitrypsin deficiency	BEAM-302 (LNP-Adenine Base Editor + hAATgRNA mRNA)	15-60 mg	phase I/II (ongoing) NCT06389877
OTC	DTX301 (scAAV8-hOTC)	1 × 10^13^ vg/kg	phase I/II (completed) phase III (ongoing) NCT05345171
	BGT-OTCD (ssAAVLK03-OTC)	6 × 10^11^ vg/kg	phase I/II (ongoing) NCT05092685
	ECUR-506 (AAV-Arcus+AAV-ssDNA donor)	1 × 10^13^ vg/kg	phase I/II (ongoing) NCT06255782

### The gateway to the *in vivo* AAV gene transfer: Advancing liver gene therapy for hemophilia

The first AAV trial for hemophilia occurred in early 2000 in subjects affected by hemophilia B.^142^ The study explored the safety of intramuscular administration of recombinant AAV2 expressing the human coagulation factor IX (hFIX) for treating hemophilia B in adult men with severe disease. It showed no toxicity up to 40 months post injection, with successful albeit very low levels of gene transfer and expression confirmed by muscle biopsies. Furthermore, the presence of pre-existing high-titer AAV antibodies did not impair vector transduction in patients' muscle tissue, suggesting the long-term safety of this approach in humans, despite its inability to achieve therapeutically effective levels of factor IX.^143^

Hemophilia treatment via the liver raised concerns about the potential for inducing an immune response such as inhibitors against the expressed coagulation factor. In 2003, the Herzog laboratory demonstrated the immunological tolerogenic capacity of hepatocytes.^144^ This study demonstrated that AAV-mediated gene therapy targeting the liver could induce immune tolerance to therapeutic antigens. In murine models, liver-derived hFIX expression effectively inhibited antibody production and attenuated T cell responses. This phenomenon was mediated through an antigen-specific immune regulation mechanism driven by CD4^+^ regulatory T cells, which exerted suppressive effects on immune activation.^144^^,^^145^ Nevertheless, in a recent paper, the same laboratory discovered that CD8^+^ T cell responses to AAV-transduced hepatocytes can occur by activating the interleukin (IL)-1 signaling. Therefore, the blocking of IL-1 pathway may help further prevent immune activation against AAV gene therapy.^146^

In the early 2000s, the first systemic delivery of rAAV was initiated. This was a phase I/II clinical trial aimed at treating patients with hemophilia B by administering an AAV2 vector expressing human factor IX from a hepatocyte specific promoter directly into the hepatic artery.^147^ This trial demonstrated that AAV2 vectors were capable of efficiently transducing human hepatocytes *in vivo*, resulting in the achievement of therapeutic levels of hFIX in individuals with severe hemophilia B. However, unlike what had been observed in all animal models treated to date, hFIX expression was transient, lasting approximately 8 weeks. Notably, the study observed an unexpected immune response—unlike those seen in preclinical animal models—characterized by an anti-AAV2 capsid cell-mediated immune elimination of hepatocytes, accompanied by a temporary, asymptomatic elevation in liver transaminases. Despite this, AAV2 infusions were well tolerated, without any evidence of acute or long-term toxicity.^147^ Further investigations sought to explain the discrepancy in AAV2-mediated transgene expression between animals and humans revealed that healthy human subjects harbor pre-existing AAV capsid-specific CD8^+^ T cells, which expand following AAV-mediated gene transfer. Additionally, the study showed that T cells activated by AAV2 responded to other AAV serotypes, suggesting that alternative serotypes may not effectively evade immune detection.^148^

The breakthrough in gene therapy for hemophilia B occurred during a trial in which patients exhibiting elevated liver enzyme levels were successfully treated with a short course of corticosteroids.^149^ This particular study investigated a gene-therapy approach for hemophilia B involving a single administration of an AAV8 vector expressing hFIX under the control of a liver-specific promoter.^150^ Six patients with severe hemophilia B were administered varying doses of the vector, initially without immunosuppressive therapy, and were followed for a period of 6–16 months. All participants demonstrated expression of hFIX at levels ranging from 2% to 11% of normal values. Six other subjects exhibited transient, mild elevations in liver enzymes; however, these increases were resolved following glucocorticoid treatment, without a reduction in hFIX expression. Long-term follow-up of a total of 10 treated patients has shown sustained levels of circulating hFIX, indicating the durability of the therapeutic effect.^151^The use of corticosteroids to mitigate the loss of transgene expression due to cell-mediated immune responses has therefore become a standard practice in *in vivo* AAV-based gene therapies.^152^

Nowadays, regulatory agencies have approved two distinct AAV-based gene therapies for the treatment of hemophilia B.^153^^,^^154^ Both therapies utilize a natural variant of clotting factor IX, known as hFIX Padua, which possesses enhanced intrinsic clotting activity compared to wild-type hFIX.^155^ The first approved therapy, Hemgenix (etranacogene dezaparvovec), is an AAV5 vector encoding hFIX Padua administered at a dose of 2 × 10^13^ genome copies per kilogram of body weight. A phase III trial evaluating its efficacy and safety demonstrated a significant reduction in annualized bleeding rates, a substantial increase in hFIX activity, and a decreased reliance on exogenous hFIX with an average reduction of 70% in annualized bleeding episodes. Notably, the therapy was effective even in participants with low pre-existing AAV5 neutralizing antibodies and was not associated with any serious treatment-related adverse events.^156^ The second therapy, Beqvez (fidanacogene elaparvovec), employs an AAVrh74 vector to deliver hFIX Padua at a dose of 5 × 10^11^ genome copies per kilogram.^157^ Long-term assessments showed a 71% reduction in the annualized bleeding rate and sustained hFIX activity. Importantly, no serious infusion-related adverse events, thrombotic complications, development of FIX inhibitors, or malignancies were observed.^158^ In general, although gene therapy for hemophilia B has been a success story, there are still limitations that plague the AAV-liver gene-therapy field in general. This includes the unpredictable wide dose-response variation between individuals and the inability to treat younger individuals.

A third liver-directed gene-therapy product, Roctavian (valoctocogene roxaparvovec), was recently approved for the treatment of hemophilia A.^159^ In a phase III study, this gene-therapy product, infused at a dose of 6 × 10^13^ vector copies per kilogram, showed an 84.5% reduction in the annualized treated bleeding rate. However, unlike what has been observed in the factor IX trials after about 2 years, the level of factor VIII tended to fall before reaching a lower steady-state level. At 2 years post infusion, no new safety concerns or serious treatment-related adverse events were observed.^160^^,^^161^ Additional, long -term efficacy data were recently disclosed by BioMarin (the pharmaceutical company that produces Roctavian) where subjects followed up for 4 to 7 years maintained therapeutic levels of hFVIII, which significantly decreased the annual bleeding rate.^162^ Another investigational gene therapy for hemophilia A, dirloctocogene samoparvovec (sponsored by Spark Therapeutics), has advanced to a phase III clinical trial. This trial represents a significant milestone as the first *in vivo* clinical study utilizing a laboratory-engineered capsid (AAV-LK03).^75^ The preceding phase I/II trial assessed four dose cohorts, ranging from 5 × 10^11^ to 2 × 10^12^ vector genomes per kilogram. While glucocorticoids were administered in some participants to address presumed immune responses, two individuals receiving the highest dose experienced a loss of hFVIII expression due to an immune-mediated reaction. However, in the remaining 16 participants, hFVIII expression persisted for over 2 years without significant declines in hFVIII activity. These participants demonstrated a 91.5% reduction in annualized bleeding rates. AAV-mediated hFVIII expression facilitated the discontinuation of prophylactic treatments with no major safety concerns reported.^163^ Nevertheless, in the clinical trial sponsored by BioMarin, which also features the longest patient follow-up data, a progressive decline in circulating hFVIII levels has been observed over time. Specifically, median levels decreased from 23% at 1 year post treatment to 7% at 4 years post treatment, raising concerns regarding the long-term durability of Roctavian.^8^ Furthermore, liver biopsies from treated patients have revealed significant variability in hFVIII transgene expression, despite consistent vector transduction levels across samples.^164^ These unanticipated challenges are currently under investigation and may have multiple underlying explanations.

The native hFVIII consists of a considerably large coding sequence that exceeds the packaging capacity of AAV vectors. Consequently, hFVIII cassettes used in both clinical and preclinical studies are truncated to remove the B-domain to fit within the AAV vector.^165^^,^^166^ This truncation introduces potential concerns, including the formation of novel immunogenic epitopes, which could provoke immune responses, and the exacerbation of the unfolded protein response (UPR) within hepatocytes, potentially leading to cellular toxicity.^167^ Moreover, it has been established that hFVIII is physiologically produced by sinusoidal endothelial cells in the liver rather than hepatocytes.^168^ As such, ectopic expression in hepatocytes may induce cellular stress responses, further reducing its therapeutic benefits. Furthermore, unlike hFIX, hFVIII does not have a natural variant with enhanced intrinsic activity, which limits the potential for dose reduction of the vector in clinical applications. Indeed, the vector doses employed in clinical trials for hemophilia A, such as those leading to the approval of Roctavian, are substantially higher than the AAV doses typically utilized for hemophilia B. Such elevated vector doses may contribute to exacerbate cell-intrinsic mechanisms that lead to the decline or loss of transgene expression.^169^ Additionally, clinical trials involving high systemic doses of AAV vectors for non-liver gene-therapy indications have demonstrated that *in vivo* administration of high AAV vector doses can induce acute liver toxicity, which, in severe cases, has resulted in patient fatalities.^170^ Recent observations have identified thrombotic microangiopathy (TMA) as an additional adverse effect associated with high doses of AAV vectors in various gene-therapy clinical trials, although no TMA cases in hemophilia treated patients have been reported so far.^171^ TMA is triggered by complement activation and, if left untreated, can lead to severe clinical manifestations.^172^

Preclinical studies are actively exploring strategies to enhance the safety and efficacy of hemophilia A gene-therapy products. In a very interesting study, scientists evaluated an alternative strategy utilizing ancestral sequence reconstruction.^173^ By leveraging available genomic sequence data for coagulation factor VIII and employing predictive models of molecular evolution, they generated protein variants with enhanced activity, stability, biosynthetic potential, and reduced susceptibility to inhibition by clinical anti-drug antibodies. Of note, this FVIII variant is currently employed in a clinical trial using hematopoietic stem cells *ex vivo* in subjects affected by hemophilia A.^174^ In this context, a recent research demonstrated that an engineered hFVIII variant,^175^ resistant to inactivation by activated protein C, can effectively normalize hemostasis at suboptimal expression levels without elevating the risk of thrombotic events in hemophilia A mouse models.^176^ These findings suggest that this innovative variant can sustain normal hFVIII activity at lower expression, thereby potentially reducing vector doses and mitigating the toxicities associated with AAV liver-mediated gene transfer. In addition, a porcine-human FVIII B-domain-deleted variant with enhanced secretion and biosynthetic activity delivered in an AAV8 vector^177^ has recently entered clinical trials. Nevertheless, such variants also raise the concern of individuals developing anti-hFVIII antibodies (inhibitors).

### Beyond hemophilia: Broadening the horizons of liver gene therapies

The promising success of AAV-mediated gene transfer in the treatment of hemophilia has paved the way toward the expansion of clinical trials, extending its application beyond liver-targeted therapies to a variety of other therapeutic indications.^4^ However, the clinical efficacy and safety of AAV-mediated liver gene therapy for metabolic disorders remains variable, reflecting a considerable heterogeneity in therapeutic outcomes.

A gene-therapy program targeting liver metabolic disorders was developed to address acute intermittent porphyria (AIP),^178^ a condition caused by porphobilinogen deaminase (PBGD) deficiency, leading to the accumulation of neurotoxic precursors and neurovisceral attacks.^179^ Following promising preclinical efficacy and safety data in animal models,^180^ a phase I clinical trial was initiated to evaluate the safety of a recombinant AAV5 vector expressing human PBGD under the control of a liver-specific promoter,^178^ at doses ranging from 5 × 10^11^ to 1.8 × 10^13^ genome copies per kilogram (vg/kg). The treatment demonstrated an acceptable safety profile, with no significant immune or cellular responses against the vector or transgene, although all patients developed neutralizing antibodies. Despite the absence of metabolic correction, as shown by unchanged delta-aminolevulinic acid (ALA) and porphobilinogen (PBG) levels and low levels of hepatic PBGD mRNA (5%–7% of wild type), some clinical improvements were observed, including reduced hospitalizations and decreased heme treatment requirements, although the true correction due to the gene therapy was not conclusively determined.^180^

A clinical trial investigating gene therapy for mucopolysaccharidosis type VI (MPS VI), a genetic disorder characterized by the accumulation of glycosaminoglycans (GAGs) due to arylsulfatase B (ARSB) deficiency,^181^ has been initiated after promising preclinical studies.^182^ In this phase I/II trial, nine patients were treated with an AAV8 delivering ARSB under a liver-specific promoter^182^ at three different doses: low (6 × 10^11^ vg/kg), intermediate (2 × 10^12^ vg/kg), and high (6 × 10^12^ vg/kg).^183^ Four patients with a modest elevation of liver enzymes post AAV administration were treated with a course of corticosteroids (prednisone). While participants in the low- and intermediate-dose cohorts did not exhibit significant clinical improvement, those in the high-dose cohort demonstrated sustained ARSB activity and stabilization of the disease, with no need for enzyme replacement therapy (ERT) for up to 4 years. Notably, one patient in the high-dose cohort resumed ERT after 2.5 years due to elevated urinary GAG concentrations, despite stable ARSB levels.^184^

A more recent study investigated the safety and efficacy of a single intravenous infusion of an AAV8 vector encoding UGT1A1^185^ enzyme in patients with Crigler-Najjar syndrome, a rare genetic liver disorder leading to severe unconjugated hyperbilirubinemia.^186^ Five patients, already receiving phototherapy (standard of care for this condition), were given either 2 × 10^12^ or 5 × 10^12^ vg/kg of the gene-therapy product.^187^ In this trial a specific immune suppressive regimen was deployed for patients. Sirolimus was adjusted to specific blood levels from 1 week before to 12 weeks after vector administration. Prednisone was started the day before AAV treatment and tapered off by week 8. If immune reactions (e.g., elevated liver enzymes) occurred, additional prednisone was given as needed. The results in the treated subjects showed no serious adverse events. Remarkably, patients receiving the higher dose achieved bilirubin levels below 300 μmol/L and did not require phototherapy for up to 78 weeks after treatment.^187^ Recently, Genethon (the sponsor of the trial) and Hansa Biopharma have announced the start of phase II study in patients with NAbs who will be pre-treated with IdeS in order to deplete the reservoir of IgG and hopefully allow for effective AAV administration.^188^ If successful, this approach could expand the eligibility criteria for AAV-based gene therapies, including pediatric and neonatal patient populations.

Ornithine transcarbamylase (OTC) deficiency, the most prevalent urea cycle disorder, results from a genetic mutation on the X chromosome affecting the OTC enzyme, which is crucial for ammonia detoxification in the liver.^189^ Ultragenyx has developed DTX301, an AAV8-based gene therapy designed to express the OTC enzyme under a liver-specific promoter.^190^ In a phase I/II trial, six of nine patients treated with a 1 × 10^13^ vg/kg dose achieved sustained metabolic control without significant adverse events.^191^ A phase III clinical trial is currently underway, enrolling up to 50 participants, who will receive a 1.7 × 10^13^ vg/kg dose.^192^^,^^193^ A new clinical trial has recently commenced in the United Kingdom for the treatment of OTC deficiency. In this phase I/II study, promising outcomes were observed in a 10-year-old patient who received the LK03-OTC vector^194^ at a dose of 6 × 10^11^ vg/kg. After 9 months of follow-up, the patient was able to discontinue ammonia-scavenger therapy without experiencing any serious adverse events.^105^^,^^195^

In a recent press release, iEcure reported the first successful application of a gene-editing approach for the treatment of OTC deficiency in a newborn patient. This strategy employed two AAV vectors: one encoding a meganuclease enzyme to induce targeted genomic DNA cleavage at the *PKSC9* locus and an ssAAV encoding an OTC expression cassette serving as a donor template for homologous recombination at the dose of 1 × 10^13^vg/kg. This dual-vector approach facilitated the efficient editing of hepatocytes, ultimately leading to phenotypic rescue of the disease at 3 months after treatment.^196^^,^^197^ Nevertheless, with this approach, the risk for prolonged meganuclease expression will require close attention.

Additionally, Ultragenyx is advancing a gene-therapy program for glycogen storage disease type Ia (GSDIa), a severe genetic disorder caused by a defect in the G6Pase-α enzyme, leading to hypoglycemia, metabolic acidosis, and liver glycogen accumulation.^198^ In a phase III trial, patients were treated with DTX401, an investigational AAV8 gene therapy designed to express the G6Pase-α enzyme under its native promoter.^191^ At a dose of 1 × 10^13^ vg/kg, the therapy significantly reduced daily cornstarch intake after 48 weeks. Secondary endpoints also showed improvements, including reductions in the frequency of cornstarch doses and better glucose regulation, albeit not curative. Safety data were consistent with earlier phase I/II studies, showing manageable, non-serious hepatic effects and no significant AAV8-related toxicities.^193^^,^^199^

Wilson disease is a rare genetic disorder caused by mutations in the *ATP7B* gene, leading to a deficiency of the ATP7B protein, which is essential for copper transport. The loss of ATP7B function results in copper accumulation in different organs, included liver and brain. Standard treatment involves life-long chelation therapy to manage copper levels.^200^ Two phase I/II liver gene-therapy studies have demonstrated promising results. In the first trial, UX701, an investigational AAV9-based gene therapy aimed at achieving stable expression of the ATP7B copper transporter was evaluated in 15 patients across three sequential dosing cohorts, receiving 5 × 10^12^, 1 × 10^13^, and 2 × 10^13^ vg/kg doses. At week 28, six patients successfully tapered off standard treatments with chelators and/or zinc. In these patients, non-ceruloplasmin-bound copper (NCC) normalized, and some showed increased ceruloplasmin-copper activity, indicating improved ATP7B function. UX701 was well tolerated, with no unexpected treatment-related adverse events or significant immunologic concerns.^201^ In another phase I/II clinical trial, VTX-801, a gene-therapy product based on a liver-tropic AAV vector expressing a shortened version of ATP7B enzyme,^202^ showed remarkable results. Two patients in cohort 1 who received a single intravenous dose of VTX-801 demonstrated favorable safety and tolerability profiles, with no serious adverse events reported. Transient, mild elevations in liver enzymes were observed. Notably, there was an increase in ceruloplasmin ferroxidase activity from baseline, indicating transgene expression in both treated patients. Additionally, liver biopsy results at 1 year post treatment revealed normalization of liver fibrosis and iron accumulation scores.^203^

Gaucher disease is a genetic disorder caused by mutations in the *GBA1* gene, leading to deficient activity of glucocerebrosidase (GCase). This deficiency results in the accumulation of toxic substrates, namely glucosylceramide (Gb1) and glucosylsphingosine (lyso-Gb1), which progressively built up in various tissues and organs, inducing inflammation and functional impairment.^204^ FLT201 is a gene-therapy product based on an engineered AAV capsid, AAVS3, expressing a rationally designed, long-acting variant of the glucocerebrosidase enzyme called GCase85.^205^ FLT201 is undergoing investigation in the phase I/II clinical trial in adults with Gaucher disease with single-dose infusion of 4.5 × 10^11^ vg/kg. The clinical trial, sponsored by Spur Therapeutics, demonstrated favorable safety and tolerability, with no infusion reactions or dose-limiting toxicities. Durable reductions in lyso-Gb1 levels were observed in patients who had persistently elevated levels despite prior ERT or substrate reduction therapy (SRT). Remarkably, significant improvements in bone marrow burden (BMB) were observed in all five patients, including those with severe bone involvement. Additionally, improvements or stabilization of spleen and liver volumes were noted more than 1 year after the withdrawal of ERT or SRT.^105^^,^^206^

Fabry disease is a lysosomal storage disorder resulting from mutations in the galactosidase alpha gene (*GLA*), leading to a deficiency in alpha-galactosidase A (α-Gal A) enzyme activity. This enzyme is essential for the catabolism of globotriaosylceramide (Gb3), and its deficiency results in the accumulation of Gb3 in various cell types. The buildup of Gb3 causes progressive damage to critical organs, including the kidneys, heart, brain, eyes, gastrointestinal tract, and skin.^207^ Sangamo therapeutics started a phase I/II trial evaluating the gene-therapy product ST-920, based on AAV6 expressing α-Gal A under a liver-specific promoter.^208^ ST-920 gene therapy at dose of 2.63 × 10^13^ vg/kg demonstrated favorable safety and tolerability in adults with symptomatic Fabry disease.^209^ Notably, no prophylactic steroids or immunomodulatory agents were administered, since no significant liver enzyme elevations were detected. The therapy exhibited durable efficacy, with supraphysiological levels of α-Gal A activity sustained for up to 36 months. All 12 subjects who discontinued ERT remained off treatment for up to 19 months. Among the eight subjects with follow-up at 12 months, 75% demonstrated an improved disease severity score compared to baseline on ERT. Notably, α-Gal A antibodies, either total or neutralizing, decreased significantly in seven subjects and became undetectable in five of them.^210^^,^^211^

The liver, known for its immunotolerant properties, has been utilized as a site for enzyme production to address disorders affecting extrahepatic tissues.^212^^,^^213^^,^^214^ A liver-directed gene-therapy clinical trial has been initiated for Pompe disease, a rare neurometabolic disorder caused by a deficiency in acid alpha-glucosidase (GAA), an enzyme essential for glycogen breakdown. The enzyme deficiency leads to glycogen accumulation, particularly in skeletal and cardiac muscles and the brain, impairing their function.^215^ While ERT has demonstrated benefits for patients affected by Pompe disease, no curative treatment currently exists.^216^ Thus, a phase I/II liver-directed gene-therapy clinical trial that utilizes an AAV8 vector encoding the GAA enzyme under a liver-specific promoter^217^ aimed to replace ERT by facilitating continuous hepatic GAA production.^218^ In the first cohort, three patients receiving a dose of 1.6 × 10^12^ vg/kg discontinued biweekly ERT after 26 weeks. Immune prophylaxis with prednisone (60 mg/day) was administered for 4 weeks and tapered thereafter. Sustained serum GAA activity was observed, with no serious treatment-related adverse events or T cell responses compromising transgene expression. Although muscle glycogen content remained unchanged by week 24, muscle GAA activity increased by week 52. Despite limited clinical symptom improvement, all patients successfully discontinued ERT, resulting in significant quality-of-life enhancements.^219^

### Liver-based mRNA and genome-editing trials

The progress in the optimization of LNPs, mRNA encapsulation, and their inherent tropism for the liver upon systemic delivery have revived their use for the treatment of hepatic diseases.^129^ A recent phase I/II study used LNP/mRNA-mediated delivery for the treatment of propionic acidemia.^220^ Propionic acidemia is a rare genetic disorder caused by defects in the propionyl-CoA carboxylase α or β (PCCA or PCCB) subunits, resulting in the accumulation of toxic metabolites and recurrent, life-threatening metabolic decompensation events.^221^ This dose-optimization study evaluated the safety and efficacy of mRNA-3927, a dual-mRNA therapy encoding PCCA and PCCB. A total of 16 participants were enrolled across five dose cohorts (from 0.3 to 0.9 mg/kg every 2 weeks), with 12 completing the dose-optimization study (2 years) and transitioning to the extension study (up to 150 weeks). A total of 346 intravenous doses of mRNA-3927 were administered. Notably, no dose-limiting toxicities were observed. Preliminary findings indicate a dose-dependent increase in mRNA-3927 exposure and a remarkable 70% reduction in the risk of metabolic decompensation events among participants.^222^ The major limitation of this approach is the need for frequent infusions making it impractical for life-long treatment.

A very promising use for the LNP/mRNA formulation to target the liver has been the delivery of CRISPR-Cas editors. In two seminal clinical trials, Intellia Therapeutics demonstrated the efficacy and the safety of deliver CRISPR-Cas9 to the liver by systemic treatment with LNPs. A first phase I/II trial aimed to treat the transthyretin amyloidosis (ATTR amyloidosis), which is a severe, progressive disorder characterized by the deposition of misfolded transthyretin (TTR) protein secreted from the liver and taken up in various tissues, particularly affecting the peripheral nerves and myocardium.^223^ The gene-therapy product NTLA-2001 is an *in vivo* gene-editing therapeutic consisting of a lipid nanoparticle delivery vehicle encapsulating mRNA encoding the CRISPR-Cas9 endonuclease and a single-guide RNA (sgRNA) specifically targeting the *TTR* gene. Preclinical *in vitro* and *in vivo* studies demonstrated durable TTR knockdown following a single NTLA-2001 dose.^224^ Subsequently, the safety and pharmacodynamic effects of escalating single doses of NTLA-2001 (0.1 and 0.3 mg/kg) were assessed in six patients with hereditary ATTR amyloidosis with polyneuropathy. Safety evaluations conducted within the first 28 days post infusion identified few adverse events, all of which were mild in severity. Dose-dependent pharmacodynamic responses were observed, with mean serum TTR reductions of 52% in the 0.1-mg/kg cohort and 87% in the 0.3-mg/kg cohort by day 28. These preliminary findings indicate that NTLA-2001 administration in a small cohort of patients with hereditary ATTR amyloidosis was well tolerated, producing significant reductions in serum TTR concentrations via targeted gene knockout.^224^ Another gene-therapy program sponsored by Intellia aimed to treat hereditary angioedema (HAE), which is a rare genetic disorder characterized by severe, unpredictable episodes of tissue swelling.^225^ NTLA-2002, an investigational *in vivo* gene-editing therapy utilizing the CRISPR-Cas9 system to target the *KLKB1* gene, encoding plasma kallikrein, was created to achieve durable control of HAE attacks after a single administration. In a phase I/II trial, doses of 25 mg, 50 mg, and 75 mg were evaluated in 10 patients.^226^ The treatment was well tolerated, with no severe or dose-limiting adverse events. Dose-dependent reductions in plasma kallikrein protein levels were observed, with mean reductions of 67% in the 25-mg cohort, 84% in the 50-mg cohort, and 95% in the 75-mg cohort. During the primary observation period (weeks 1–16), the mean reductions in monthly angioedema attack frequency were 91% (25 mg), 97% (50 mg), and 80% (75 mg). These results demonstrate NTLA-2002’s potential for durable efficacy and safety in HAE management.^227^ Furthermore, Intellia/Regeneron Pharmaceuticals has initiated a clinical trial targeting pediatric patients with hemophilia B. The trial employs a combination of LNPs delivering mRNA encoding CRISPR-Cas9 and an ssAAV vector carrying donor DNA encoding the hFIX cDNA.^228^ The objective of this study is to achieve targeted integration of the hFIX cDNA into an intron of the *ALBUMIN* locus in actively proliferating hepatocytes of young patients, thereby mitigating the loss of the episomal AAV genome. If successful, it would pave the way for treating individuals with this disease at the time of diagnosis shortly after their birth.

Interestingly, Beam Therapeutics recently reported encouraging preliminary findings from their phase I/II clinical trial in individuals with alpha-1 antitrypsin deficiency (AATD). In this study, patients received LNP-delivered base editors targeting hepatocytes to correct the PiZ mutation. This genetic correction resulted in a reduction of the pathogenic Z-AAT protein and a concomitant increase in the expression of the functional M-AAT protein.^229^

Nevertheless, there have already been two clinical trials attempting to precisely insert a missing transgene into the highly transcribed hepatocyte specific *ALBUMIN* locus. The first was Sangamo’s SB-913, a ZFN-mediated gene-editing therapy for treating mucopolysaccharidosis II (MPS II) or Hunter’s syndrome. MPS II is a progressive inherited lysosomal storage disorder caused by lack of iduronate-2-sulfatase enzyme, which degrades glycosaminoglycans.^230^ This approach utilized multiple AAVs, one of which encoded the nuclease and the second the therapeutic transgene coding sequence. This approach was not successful.^231^

The second, hLB-001, involved treatment of children with methylmalonic acidemia, resulting from the inability to metabolize branch-chain amino acids due to a lack of methylmalonyl-CoA mutase (MMA).^232^ MMA-positive hepatocytes show a selective advantage and expand in mouse models of the disease.^233^ The trial made use of a nuclease-free mediated insertion of the therapeutic coding sequences into the 3′ end of the *ALBUMIN* locus via homologous recombination to create a locus that transcribes an mRNA that encodes both the albumin protein with a P2A ribosomal skipping site followed by the inserted coding region,^234^ in this case MMA.^235^ Thus, in targeted *ALBUMIN* alleles, one transcript makes two proteins and the P2A (tag)-ALBUMIN, the latter of which can be quantified by ELISA. Four patients were enrolled in the trial, and while P2A-ALBUMIN was detected, and even with increasing P2A-ALBUMIN expression, the amount of genetic reconstitution was not high enough to provide a detectable clinical benefit in patients.^134^ Nonetheless, it did establish that precise gene insertion of a therapeutic coding sequence was possible in humans.

### Conclusions

**Figure 3:**
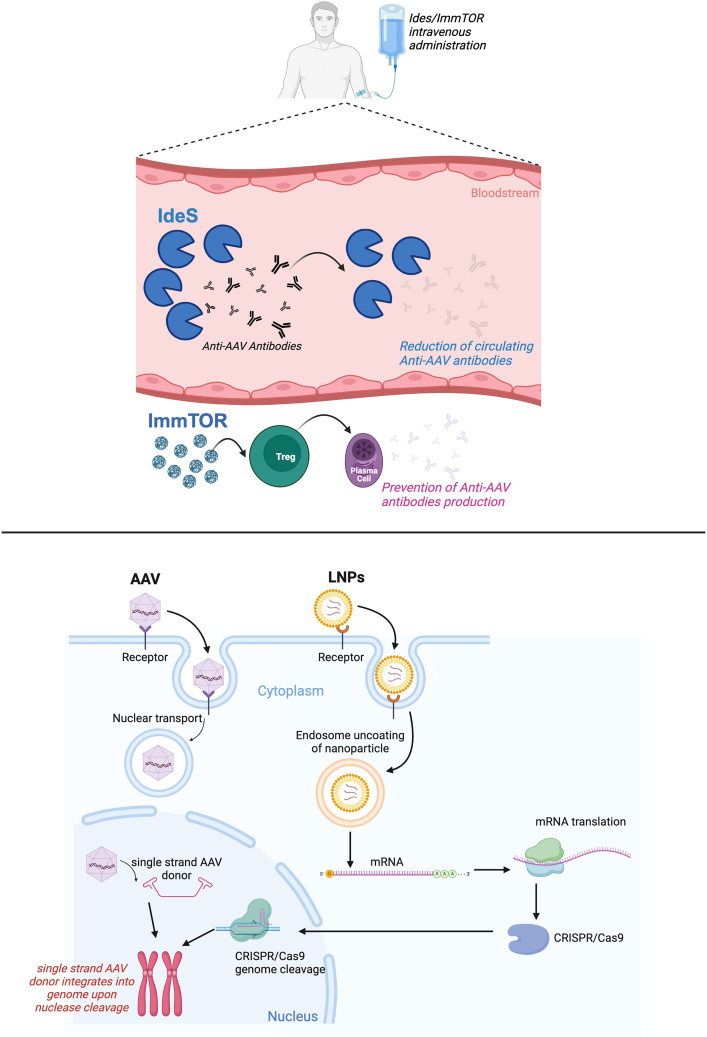
Innovative strategies currently under investigation in clinical trials to address the limitations associated with the use of AAV vectors for liver gene therapy (Top) Mitigating immune responses against AAV vectors. Intravenous administration of IdeS facilitates the cleavage of pre-existing anti-AAV antibodies within the bloodstream. This enzymatic activity generates antibody fragments that are incapable of neutralizing AAV vectors, thereby reducing circulating antibody titers. This reduction enhances the efficacy of AAV vector delivery in seropositive individuals and allows re-administration in patients who have previously been treated with AAV-based gene therapies. Treatment with ImmTOR nanoparticles promotes the induction of regulatory T cells (Tregs), which suppress the activity of plasma cells responsible for producing anti-AAV antibodies. This dual action minimizes both existing and newly generated anti-AAV antibodies, enabling re-dosing and successful AAV delivery in seropositive subjects. (Bottom) Mechanisms of AAV and LNP platforms for CRISPR-Cas9-mediated genome editing in liver gene therapy. LNPs bind to specific cell surface receptors and are internalized via endocytosis. Once within the cytoplasm, LNPs facilitate the release of mRNA encoding CRISPR-Cas9 components through endosomal escape mechanisms. The released mRNA is subsequently translated into the Cas9 protein, which translocates to the nucleus to induce precise genome cleavage at the targeted locus. Concurrently, AAV vectors bind to cell surface receptors, are transported from the cytoplasm into the nucleus, and deliver a single-stranded DNA (ssDNA) donor template. This donor template integrates into the genome at the site of CRISPR-Cas9-induced DSBs via homology-directed repair (HDR). This dual delivery strategy is particularly advantageous for addressing AAV genome loss in pediatric patients, where continuous liver growth poses a challenge to sustained gene-editing efficacy.

More than 20 years have passed since the first AAV-based gene-therapy product was administered to patients with hemophilia B.^142^ Over the past two decades, the promise of a one-time, curative treatment for liver diseases has been realized, culminating in the approval of three gene-therapy products. The clinical outcomes summarized in this review have been transformative and changed the lives of many patients affected by a variety of genetic disorders. Thus, we expect continued approved AAV liver-targeted gene therapies will reach the market in the near future. However, several challenges remain to be addressed to enhance the safety and efficacy of these therapies. Even though there are many examples of clinical improvement, most of these gene-therapy trials have not been curative. In addition, a significant concern is the occurrence of AAV-related acute toxicities. The most severe toxicities have included severe liver failure, although these occurred in non-liver trials such as spinal muscular atrophy (SMA),^170^ Duchenne muscular dystrophy (DMD), and X-linked myotubular myopathy (XLMTM)^236^ where high vector doses (>1 × 10^14^ vg/kg) are required.^169^^,^^237^ In some instances, these adverse events have tragically resulted in patient fatalities.^238^^,^^239^^,^^240^ Consequently, the development of more potent AAV vectors capable of efficiently transducing target cells is imperative to reduce the required dosage and minimize vector-mediated toxicity. In the case of liver-directed gene therapies, species-specific barriers hinder the translation of preclinical successes into human applications. While natural AAV vectors such as AAV8 can transduce nearly 100% of hepatocytes in rodents with extraordinarily high transgene expression, equivalent efficiency has not been achieved in NHPs or humans. Additionally, in cohorts of patients treated with the same AAV vector, there has been substantial variability in dose response independent of pre-existing immunity. At this time, the reason for these variable responses may be multi-factorial and warrant further investigations. Thus, improving the AAV potency will enable dose reductions and enhance vector manufacturing. At present, the cost of AAV vectors for clinical trials is exorbitant and unsustainable long term with the production costs in the range of $500,000–$1 million and commercialization price between 1 million and 3 million dollars.^241^^,^^242^ Moreover, the lack of standardized AAV manufacturing processes exacerbates this problem. For instance, the three approved gene therapies employ distinct production methods, leading to further increases in the costs, which are already very high. Importantly, this economic burden limits equitable access to these treatments on a worldwide scale.^243^ Another major obstacle to the broader clinical application of AAV-based gene therapies is the exclusion of specific patient populations, primarily due to two factors: (1) pediatric patients, in which growing and dividing liver cells prevent stable transgene expression, and (2) the presence of high titers of NAbs that preclude AAV administration. Addressing these issues could significantly expand the pool of eligible patients. For example, strategies to deplete NAbs may enable the initial dosing and potential for re-dosing AAV vectors (Figure 3A), particularly in cases where the first treatment does not yield sufficient clinical benefit. In pediatric patients, an initial treatment during infancy could prevent irreversible disease progression, while a subsequent administration in adulthood might offer long-term curative outcomes. Additionally, gene-editing approaches hold promise for pediatric applications by enabling permanent genome modifications, thus circumventing issues related to AAV genome dilution during organ growth (Figure 3B). These outcomes in theory require transient gene-editor expression (e.g., LNP-mediated delivery of mRNA) in conjunction with AAV-mediated delivery of the donor DNA template. Furthermore, the future clinical deployment of LV vectors could offer alternative therapeutic options, particularly for younger patients.

In conclusion, the progress achieved in the field of AAV-based gene therapy over the past two decades has been remarkable, with clinical outcomes demonstrating efficacy in addressing previously untreatable diseases. Continued advancements are occurring at an extraordinary pace, and it is anticipated that many of the remaining challenges in AAV-based gene therapy for liver disorders will be resolved within the next decade. These innovations have the potential to pave the way for treatments for a broad spectrum of genetic and acquired diseases. However, while not every scientific advancement will lead to a treatment, many will find a clinical niche based on the underlying disease pathophysiology.

## Acknowledgments

This work was supported by 10.13039/100000002NIH grants R01HL064274 and R01AI116698. The figures were generated using Biorender.com.

## Author contributions

F.P. and M.A.K. wrote the manuscript. F.P. generated the table and figures.

## Declaration of interests

Stanford owns the patents related to the AAV-LK03 vector for which M.A.K. is an inventor.

## References

[1] High K., Roncarolo M.G. (2019). Gene Therapy Gene Therapy. N. Engl. J. Med..

[2] Kay M.A. (2011). State-of-the-art gene-based therapies: the road ahead. Nat. Rev. Genet..

[3] Schulze R.J., Schott M.B., Casey C.A., Tuma P.L., McNiven M.A. (2019). The cell biology of the hepatocyte: A membrane trafficking machine. J. Cell Biol..

[4] Zabaleta N., Unzu C., Weber N.D., Gonzalez-Aseguinolaza G. (2023). Gene therapy for liver diseases — progress and challenges. Nat. Rev. Gastroenterol. Hepatol..

[5] Wang D., Tai P.W.L., Gao G. (2019). Adeno-associated virus vector as a platform for gene therapy delivery. Nat. Rev. Drug Discov..

[6] Research, C. for B.E. (2024).

[7] Costa-Verdera H., Unzu C., Valeri E., Adriouch S., González Aseguinolaza G., Mingozzi F., Kajaste-Rudnitski A. (2023). Understanding and Tackling Immune Responses to Adeno-Associated Viral Vectors. Hum. Gene Ther..

[8] Pierce G.F., Fong S., Long B.R., Kaczmarek R. (2024). Deciphering conundrums of adeno-associated virus liver-directed gene therapy: focus on hemophilia. J. Thromb. Haemost..

[9] A phase 1/2 Safety and Efficacy Study of TAK-754 Gene Therapy: The Challenge of Achieving Durable Factor VIII Expression in Haemophilia A Clinical Trials - Chapin - Haemophilia - Wiley Online Library https://onlinelibrary.wiley.com/doi/10.1111/hae.15121. 10.1111/hae.15121 PMC11780198 39716875

[10] Sabatino D.E., Bushman F.D., Chandler R.J., Crystal R.G., Davidson B.L., Dolmetsch R., Eggan K.C., Gao G., Gil-Farina I., Kay M.A. (2022). Evaluating the state of the science for adeno-associated virus integration: An integrated perspective. Mol. Ther..

[11] Li C., Samulski R.J. (2020). Engineering adeno-associated virus vectors for gene therapy. Nat. Rev. Genet..

[12] Earley J., Piletska E., Ronzitti G., Piletsky S. (2023). Evading and overcoming AAV neutralization in gene therapy. Trends Biotechnol..

[13] Whiteley L.O. (2023). An Overview of Nonclinical and Clinical Liver Toxicity Associated With AAV Gene Therapy. Toxicol. Pathol..

[14] Ables C., Jaramillo C., Wood E.L., Stern S., Alashari M., Book L., Butterfield R.J. (2024). Subacute liver injury in two young infants following gene replacement therapy for spinal muscular atrophy. Mol. Ther. Methods Clin. Dev..

[15] Sorge J., Wright D., Erdman V.D., Cutting A.E. (1984). Amphotropic retrovirus vector system for human cell gene transfer. Mol. Cell. Biol..

[16] Chowdhury J.R., Grossman M., Gupta S., Chowdhury N.R., Baker J.R., Wilson J.M. (1991). Long-term improvement of hypercholesterolemia after ex vivo gene therapy in LDLR-deficient rabbits. Science.

[17] Kay M.A., Baley P., Rothenberg S., Leland F., Fleming L., Ponder K.P., Liu T., Finegold M., Darlington G., Pokorny W. (1992). Expression of human alpha 1-antitrypsin in dogs after autologous transplantation of retroviral transduced hepatocytes. Proc. Natl. Acad. Sci. USA.

[18] Grossman M., Rader D.J., Muller D.W., Kolansky D.M., Kozarsky K., Clark B.J., Stein E.A., Lupien P.J., Brewer H.B., Raper S.E. (1995). A pilot study of ex vivo gene therapy for homozygous familial hypercholesterolaemia. Nat. Med..

[19] Nakai H., Yant S.R., Storm T.A., Fuess S., Meuse L., Kay M.A. (2001). Extrachromosomal recombinant adeno-associated virus vector genomes are primarily responsible for stable liver transduction in vivo. J. Virol..

[20] Overturf K., Al-Dhalimy M., Tanguay R., Brantly M., Ou C.-N., Finegold M., Grompe M. (1996). Hepatocytes corrected by gene therapy are selected in vivo in a murine model of hereditary tyrosinaemia type I. Nat. Genet..

[21] Montini E., Held P.K., Noll M., Morcinek N., Al-Dhalimy M., Finegold M., Yant S.R., Kay M.A., Grompe M. (2002). In vivo correction of murine tyrosinemia type I by DNA-mediated transposition. Mol. Ther..

[22] Held P.K., Olivares E.C., Aguilar C.P., Finegold M., Calos M.P., Grompe M. (2005). In vivo correction of murine hereditary tyrosinemia type I by phiC31 integrase-mediated gene delivery. Mol. Ther..

[23] Liu M.L., Winther B.L., Kay M.A. (1996). Pseudotransduction of hepatocytes by using concentrated pseudotyped vesicular stomatitis virus G glycoprotein (VSV-G)-Moloney murine leukemia virus-derived retrovirus vectors: comparison of VSV-G and amphotropic vectors for hepatic gene transfer. J. Virol..

[24] Cardoso J.E., Branchereau S., Jeyaraj P.R., Houssin D., Danos O., Heard J.M. (1993). In Situ Retrovirus-Mediated Gene Transfer into Dog Liver. Hum. Gene Ther..

[25] Kay M.A., Rothenberg S., Landen C.N., Bellinger D.A., Leland F., Toman C., Finegold M., Thompson A.R., Read M.S., Brinkhous K.M. (1993). In Vivo Gene Therapy of Hemophilia B: Sustained Partial Correction in Factor IX-Deficient Dogs. Science.

[26] Powell J.S., Ragni M.V., White G.C., Lusher J.M., Hillman-Wiseman C., Moon T.E., Cole V., Ramanathan-Girish S., Roehl H., Sajjadi N. (2003). Phase 1 trial of FVIII gene transfer for severe hemophilia A using a retroviral construct administered by peripheral intravenous infusion. Blood.

[27] Naldini L., Blömer U., Gallay P., Ory D., Mulligan R., Gage F.H., Verma I.M., Trono D. (1996). In vivo gene delivery and stable transduction of nondividing cells by a lentiviral vector. Science.

[28] Naldini L. (1998). Lentiviruses as gene transfer agents for delivery to non-dividing cells. Curr. Opin. Biotechnol..

[29] Park F., Ohashi K., Chiu W., Naldini L., Kay M.A. (2000). Efficient lentiviral transduction of liver requires cell cycling in vivo. Nat. Genet..

[30] Bulcha J.T., Wang Y., Ma H., Tai P.W.L., Gao G. (2021). Viral vector platforms within the gene therapy landscape. Signal Transduct. Target. Ther..

[31] Cantore A., Fraldi A., Meneghini V., Gritti A. (2021). In vivo Gene Therapy to the Liver and Nervous System: Promises and Challenges. Front. Med..

[32] Cesana D., Ranzani M., Volpin M., Bartholomae C., Duros C., Artus A., Merella S., Benedicenti F., Sergi Sergi L., Sanvito F. (2014). Uncovering and dissecting the genotoxicity of self-inactivating lentiviral vectors in vivo. Mol. Ther..

[33] Annoni A., Goudy K., Akbarpour M., Naldini L., Roncarolo M.G. (2013). Immune responses in liver-directed lentiviral gene therapy. Transl. Res..

[34] Carbonaro-Sarracino D.A., Tarantal A.F., Lee C.C.I., Kaufman M.L., Wandro S., Jin X., Martinez M., Clark D.N., Chun K., Koziol C. (2020). Dosing and Re-Administration of Lentiviral Vector for In Vivo Gene Therapy in Rhesus Monkeys and ADA-Deficient Mice. Mol. Ther. Methods Clin. Dev..

[35] Milani M., Canepari C., Assanelli S., Merlin S., Borroni E., Starinieri F., Biffi M., Russo F., Fabiano A., Zambroni D. (2024). GP64-pseudotyped lentiviral vectors target liver endothelial cells and correct hemophilia A mice. EMBO Mol. Med..

[36] Milani M., Annoni A., Bartolaccini S., Biffi M., Russo F., Di Tomaso T., Raimondi A., Lengler J., Holmes M.C., Scheiflinger F. (2017). Genome editing for scalable production of alloantigen-free lentiviral vectors for in vivo gene therapy. EMBO Mol. Med..

[37] Milani M., Annoni A., Moalli F., Liu T., Cesana D., Calabria A., Bartolaccini S., Biffi M., Russo F., Visigalli I. (2019). Phagocytosis-shielded lentiviral vectors improve liver gene therapy in nonhuman primates. Sci. Transl. Med..

[38] Milani M., Canepari C., Liu T., Biffi M., Russo F., Plati T., Curto R., Patarroyo-White S., Drager D., Visigalli I. (2022). Liver-directed lentiviral gene therapy corrects hemophilia A mice and achieves normal-range factor VIII activity in non-human primates. Nat. Commun..

[39] Cantore A., Ranzani M., Bartholomae C.C., Volpin M., Valle P.D., Sanvito F., Sergi L.S., Gallina P., Benedicenti F., Bellinger D. (2015). Liver-directed lentiviral gene therapy in a dog model of hemophilia B. Sci. Transl. Med..

[40] Stratford-Perricaudet L.D., Levrero M., Chasse J.-F., Perricaudet M., Briand P. (1990). Evaluation of the Transfer and Expression in Mice of an Enzyme-Encoding Gene Using a Human Adenovirus Vector. Hum. Gene Ther..

[41] Kay M.A., Landen C.N., Rothenberg S.R., Taylor L.A., Leland F., Wiehle S., Fang B., Bellinger D., Finegold M., Thompson A.R. (1994). In vivo hepatic gene therapy: complete albeit transient correction of factor IX deficiency in hemophilia B dogs. Proc. Natl. Acad. Sci. USA.

[42] Parks R.J., Chen L., Anton M., Sankar U., Rudnicki M.A., Graham F.L. (1996). A helper-dependent adenovirus vector system: Removal of helper virus by Cre-mediated excision of the viral packaging signal. Proc. Natl. Acad. Sci..

[43] Mitani K., Graham F.L., Caskey C.T., Kochanek S. (1995). Rescue, propagation, and partial purification of a helper virus-dependent adenovirus vector. Proc. Natl. Acad. Sci..

[44] Lehrman S. (1999). Virus treatment questioned after gene therapy death. Nature.

[45] Brunetti-Pierri N., Liou A., Patel P., Palmer D., Grove N., Finegold M., Piccolo P., Donnachie E., Rice K., Beaudet A. (2012). Balloon catheter delivery of helper-dependent adenoviral vector results in sustained, therapeutic hFIX expression in rhesus macaques. Mol. Ther..

[46] Morral N., O’Neal W., Rice K., Leland M., Kaplan J., Piedra P.A., Zhou H., Parks R.J., Velji R., Aguilar-Córdova E. (1999). Administration of helper-dependent adenoviral vectors and sequential delivery of different vector serotype for long-term liver-directed gene transfer in baboons. Proc. Natl. Acad. Sci..

[47] Miyanohara A., Johnson P.A., Elam R.L., Dai Y., Witztum J.L., Verma I.M., Friedmann T. (1992). Direct gene transfer to the liver with herpes simplex virus type 1 vectors: transient production of physiologically relevant levels of circulating factor IX. New Biol..

[48] Strayer D.S. (1999). Gene therapy using SV40-derived vectors: what does the future hold?. J. Cell. Physiol..

[49] Wu G.Y., Wu C.H. (1993). Liver-directed gene delivery. Adv. Drug Deliv. Rev..

[50] Liu F., Song Y., Liu D. (1999). Hydrodynamics-based transfection in animals by systemic administration of plasmid DNA. Gene Ther..

[51] Zhang G., Budker V., Wolff J.A. (1999). High levels of foreign gene expression in hepatocytes after tail vein injections of naked plasmid DNA. Hum. Gene Ther..

[52] Khorsandi S.E., Bachellier P., Weber J.C., Greget M., Jaeck D., Zacharoulis D., Rountas C., Helmy S., Helmy A., Al-Waracky M. (2008). Minimally invasive and selective hydrodynamic gene therapy of liver segments in the pig and human. Cancer Gene Ther..

[53] Chen Z.-Y., He C.-Y., Ehrhardt A., Kay M.A. (2003). Minicircle DNA vectors devoid of bacterial DNA result in persistent and high-level transgene expression in vivo. Mol. Ther..

[54] Yant S.R., Meuse L., Chiu W., Ivics Z., Izsvak Z., Kay M.A. (2000). Somatic integration and long-term transgene expression in normal and haemophilic mice using a DNA transposon system. Nat. Genet..

[55] Tipanee J., VandenDriessche T., Chuah M.K. (2017). Transposons: Moving Forward from Preclinical Studies to Clinical Trials. Hum. Gene Ther..

[56] Atchison R.W., Casto B.C., Hammon W.M.D. (1965). Adenovirus-Associated Defective Virus Particles. Science.

[57] Hermonat P.L., Muzyczka N. (1984). Use of adeno-associated virus as a mammalian DNA cloning vector: transduction of neomycin resistance into mammalian tissue culture cells. Proc. Natl. Acad. Sci..

[58] Kotin R.M., Linden R.M., Berns K.I. (1992). Characterization of a preferred site on human chromosome 19q for integration of adeno-associated virus DNA by non-homologous recombination. EMBO J..

[59] Snyder R.O., Miao C., Meuse L., Tubb J., Donahue B.A., Lin H.F., Stafford D.W., Patel S., Thompson A.R., Nichols T. (1999). Correction of hemophilia B in canine and murine models using recombinant adeno-associated viral vectors. Nat. Med..

[60] Mount J.D., Herzog R.W., Tillson D.M., Goodman S.A., Robinson N., McCleland M.L., Bellinger D., Nichols T.C., Arruda V.R., Lothrop C.D., High K.A. (2002). Sustained phenotypic correction of hemophilia B dogs with a factor IX null mutation by liver-directed gene therapy. Blood.

[61] Snyder R.O., Miao C.H., Patijn G.A., Spratt S.K., Danos O., Nagy D., Gown A.M., Winther B., Meuse L., Cohen L.K. (1997). Persistent and therapeutic concentrations of human factor IX in mice after hepatic gene transfer of recombinant AAV vectors. Nat. Genet..

[62] Gao G.-P., Alvira M.R., Wang L., Calcedo R., Johnston J., Wilson J.M. (2002). Novel adeno-associated viruses from rhesus monkeys as vectors for human gene therapy. Proc. Natl. Acad. Sci. USA.

[63] Grimm D., Lee J.S., Wang L., Desai T., Akache B., Storm T.A., Kay M.A. (2008). In Vitro and In Vivo Gene Therapy Vector Evolution via Multispecies Interbreeding and Retargeting of Adeno-Associated Viruses. J. Virol..

[64] Lam A.K., Frabutt D., Li L., Xiao W. (2021). Chemical Modifications of the Capsid for Redirecting and Improving the Efficacy of Adeno-Associated Virus Vectors. Hum. Gene Ther..

[65] Puzzo F., Zhang C., Powell Gray B., Zhang F., Sullenger B.A., Kay M.A. (2023). Aptamer-programmable adeno-associated viral vectors as a novel platform for cell-specific gene transfer. Mol. Ther. Nucleic Acids.

[66] Asokan A., Schaffer D.V., Samulski R.J. (2012). The AAV vector toolkit: poised at the clinical crossroads. Mol. Ther..

[67] Raguram A., Banskota S., Liu D.R. (2022). Therapeutic *in vivo* delivery of gene editing agents. Cell.

[68] Banskota S., Raguram A., Suh S., Du S.W., Davis J.R., Choi E.H., Wang X., Nielsen S.C., Newby G.A., Randolph P.B. (2022). Engineered virus-like particles for efficient in vivo delivery of therapeutic proteins. Cell.

[69] Raguram A., An M., Chen P.Z., Liu D.R. (2024). Directed evolution of engineered virus-like particles with improved production and transduction efficiencies. Nat. Biotechnol..

[70] Decades after a tragic failure, gene therapy successfully treats a rare liver disease https://www.science.org/content/article/decades-after-tragic-failure-gene-therapy-successfully-treats-rare-liver-disease.

[71] Palaschak B., Herzog R.W., Markusic D.M., Castle M.J. (2019). Adeno-Associated Virus Vectors: Design and Delivery.

[72] Colón-Thillet R., Jerome K.R., Stone D. (2021). Optimization of AAV vectors to target persistent viral reservoirs. Virol. J..

[73] Klenchin V.A., Clark N.M., Keles N.K., Capuano S., Mason R., Gao G., Broman A., Kose E., Immonen T.T., Fennessey C.M. (2025). Adeno-associated viral delivery of Env-specific antibodies prevents SIV rebound after discontinuing antiretroviral therapy. Sci. Immunol..

[74] Joshi L.R., Gálvez N.M.S., Ghosh S., Weiner D.B., Balazs A.B. (2023). Delivery platforms for broadly neutralizing antibodies. Curr. Opin. HIV AIDS.

[75] Lisowski L., Dane A.P., Chu K., Zhang Y., Cunningham S.C., Wilson E.M., Nygaard S., Grompe M., Alexander I.E., Kay M.A. (2014). Selection and evaluation of clinically relevant AAV variants in a xenograft liver model. Nature.

[76] Gonzalez-Sandoval A., Pekrun K., Tsuji S., Zhang F., Hung K.L., Chang H.Y., Kay M.A. (2023). The AAV capsid can influence the epigenetic marking of rAAV delivered episomal genomes in a species dependent manner. Nat. Commun..

[77] Grompe M., Strom S. (2013). Mice with human livers. Gastroenterology.

[78] Kim J.-J., Kurial S.N.T., Choksi P.K., Nunez M., Lunow-Luke T., Bartel J., Driscoll J., Her C.L., Dhillon S., Yue W. (2025). AAV capsid prioritization in normal and steatotic human livers maintained by machine perfusion. Nat. Biotechnol..

[79] Cabanes-Creus M., Liao S.H.Y., Gale Navarro R., Knight M., Nazareth D., Lau N.-S., Ly M., Zhu E., Roca-Pinilla R., Bugallo Delgado R. (2024). Harnessing whole human liver ex situ normothermic perfusion for preclinical AAV vector evaluation. Nat. Commun..

[80] Catalyzing the Development and Use of Novel Alternative Methods. https://www.nih.gov/about-nih/who-we-are/nih-director/statements/statement-catalyzing-development-novel-alternatives-methods.

[81] Kim J., Koo B.-K., Knoblich J.A. (2020). Human organoids: model systems for human biology and medicine. Nat. Rev. Mol. Cell Biol..

[82] Afonso M.B., Marques V., van Mil S.W.C., Rodrigues C.M.P. (2024). Human liver organoids: From generation to applications. Hepatol. Baltim. Md..

[83] Prior N., Inacio P., Huch M. (2019). Liver organoids: from basic research to therapeutic applications. Gut.

[84] Ma C., Peng Y., Li H., Chen W. (2021). Organ-on-a-Chip: A New Paradigm for Drug Development. Trends Pharmacol. Sci..

[85] Liu J., Du Y., Xiao X., Tan D., He Y., Qin L. (2024). Construction of in vitro liver-on-a-chip models and application progress. Biomed. Eng. Online.

[86] Clavien P.-A., Dutkowski P., Mueller M., Eshmuminov D., Bautista Borrego L., Weber A., Muellhaupt B., Sousa Da Silva R.X., Burg B.R., Rudolf von Rohr P. (2022). Transplantation of a human liver following 3 days of ex situ normothermic preservation. Nat. Biotechnol..

[87] Paulk N.K., Pekrun K., Zhu E., Nygaard S., Li B., Xu J., Chu K., Leborgne C., Dane A.P., Haft A. (2018). Bioengineered AAV Capsids with Combined High Human Liver Transduction In Vivo and Unique Humoral Seroreactivity. Mol. Ther..

[88] Dhungel B.P., Bailey C.G., Rasko J.E.J. (2021). Journey to the Center of the Cell: Tracing the Path of AAV Transduction. Trends Mol. Med..

[89] Gonzalez T.J., Simon K.E., Blondel L.O., Fanous M.M., Roger A.L., Maysonet M.S., Devlin G.W., Smith T.J., Oh D.K., Havlik L.P. (2022). Cross-species evolution of a highly potent AAV variant for therapeutic gene transfer and genome editing. Nat. Commun..

[90] Boutin S., Monteilhet V., Veron P., Leborgne C., Benveniste O., Montus M.F., Masurier C. (2010). Prevalence of serum IgG and neutralizing factors against adeno-associated virus (AAV) types 1, 2, 5, 6, 8, and 9 in the healthy population: Implications for gene therapy using AAV vectors. Hum. Gene Ther..

[91] Costa Verdera H., Kuranda K., Mingozzi F. (2020). AAV Vector Immunogenicity in Humans: A Long Journey to Successful Gene Transfer. Mol. Ther..

[92] Human Immunoglobulin Inhibits Liver Transduction by AAV Vectors at Low AAV2 Neutralizing Titers in SCID Mice | Blood | American Society of Hematology https://ashpublications.org/blood/article/107/5/1810/133412/Human-immunoglobulin-inhibits-liver-transduction. 10.1182/blood-2005-08-3229 16249376

[93] Schulz M., Levy D.I., Petropoulos C.J., Bashirians G., Winburn I., Mahn M., Somanathan S., Cheng S.H., Byrne B.J. (2023). Binding and neutralizing anti-AAV antibodies: Detection and implications for rAAV-mediated gene therapy. Mol. Ther..

[94] Colella P., Ronzitti G., Mingozzi F. (2018). Emerging Issues in AAV-Mediated *In Vivo* Gene Therapy. Mol. Ther. Methods Clin. Dev..

[95] Potter R.A., Peterson E.L., Griffin D., Cooper Olson G., Lewis S., Cochran K., Mendell J.R., Rodino-Klapac L.R. (2024). Use of plasmapheresis to lower anti-AAV antibodies in nonhuman primates with pre-existing immunity to AAVrh74. Mol. Ther. Methods Clin. Dev..

[96] Orlowski A., Katz M.G., Gubara S.M., Fargnoli A.S., Fish K.M., Weber T. (2020). Successful Transduction with AAV Vectors after Selective Depletion of Anti-AAV Antibodies by Immunoadsorption. Mol. Ther. Methods Clin. Dev..

[97] Mokrzycki M.H., Kaplan A.A. (1994). Therapeutic Plasma Exchange: Complications and Management. Am. J. Kidney Dis..

[98] Lu J., Zhang L., Xia C., Tao Y. (2019). Complications of therapeutic plasma exchange. Medicine (Baltimore).

[99] von Pawel-Rammingen U., Johansson B.P., Björck L. (2002). IdeS, a novel streptococcal cysteine proteinase with unique specificity for immunoglobulin G. EMBO J..

[100] Jordan S.C., Lorant T., Choi J., Kjellman C., Winstedt L., Bengtsson M., Zhang X., Eich T., Toyoda M., Eriksson B.-M. (2017). IgG Endopeptidase in Highly Sensitized Patients Undergoing Transplantation. N. Engl. J. Med..

[101] Takahashi R., Yuki N. (2015). Streptococcal IdeS: therapeutic potential for Guillain–Barré syndrome. Sci. Rep..

[102] Leborgne C., Barbon E., Alexander J.M., Hanby H., Delignat S., Cohen D.M., Collaud F., Muraleetharan S., Lupo D., Silverberg J. (2020). IgG-cleaving endopeptidase enables in vivo gene therapy in the presence of anti-AAV neutralizing antibodies. Nat. Med..

[103] Smith T.J., Elmore Z.C., Fusco R.M., Hull J.A., Rosales A., Martinez M., Tarantal A.F., Asokan A. (2024). Engineered IgM and IgG cleaving enzymes for mitigating antibody neutralization and complement activation in AAV gene transfer. Mol. Ther..

[104] Cao M., Katial R., Liu Y., Lu X., Gu Q., Chen C., Liu K., Zhu Z., Marshall M.R., Yu Y., Wang Z. (2025). Safety, efficacy, and immunogenicity of a novel IgG degrading enzyme (KJ103): results from two randomised, blinded, phase 1 clinical trials. Gene Ther..

[105] ESGCT 31st Annual Congress In collaboration with SITGEC Rome, Italy October 22–25, 2024 Abstracts (2025). Hum. Gene Ther..

[106] Maldonado R.A., LaMothe R.A., Ferrari J.D., Zhang A.-H., Rossi R.J., Kolte P.N., Griset A.P., O’Neil C., Altreuter D.H., Browning E. (2015). Polymeric synthetic nanoparticles for the induction of antigen-specific immunological tolerance. Proc. Natl. Acad. Sci..

[107] Ilyinskii P.O., Michaud A.M., Roy C.J., Rizzo G.L., Elkins S.L., Capela T., Chowdhury A.C., Leung S.S., Kishimoto T.K. (2021). Enhancement of liver-directed transgene expression at initial and repeat doses of AAV vectors admixed with ImmTOR nanoparticles. Sci. Adv..

[108] Meliani A., Boisgerault F., Hardet R., Marmier S., Collaud F., Ronzitti G., Leborgne C., Costa Verdera H., Simon Sola M., Charles S. (2018). Antigen-selective modulation of AAV immunogenicity with tolerogenic rapamycin nanoparticles enables successful vector re-administration. Nat. Commun..

[109] Horiuchi M., Hinderer C.J., Shankle H.N., Hayashi P.M., Chichester J.A., Kissel C., Bell P., Dyer C., Wilson J.M. (2023). Neonatal Fc Receptor Inhibition Enables Adeno-Associated Virus Gene Therapy Despite Pre-Existing Humoral Immunity. Hum. Gene Ther..

[110] Nakai H., Montini E., Fuess S., Storm T.A., Grompe M., Kay M.A. (2003). AAV serotype 2 vectors preferentially integrate into active genes in mice. Nat. Genet..

[111] Donsante A., Miller D.G., Li Y., Vogler C., Brunt E.M., Russell D.W., Sands M.S. (2007). AAV vector integration sites in mouse hepatocellular carcinoma. Science.

[112] Chandler R.J., LaFave M.C., Varshney G.K., Trivedi N.S., Carrillo-Carrasco N., Senac J.S., Wu W., Hoffmann V., Elkahloun A.G., Burgess S.M., Venditti C.P. (2015). Vector design influences hepatic genotoxicity after adeno-associated virus gene therapy. J. Clin. Invest..

[113] Dalwadi D.A., Calabria A., Tiyaboonchai A., Posey J., Naugler W.E., Montini E., Grompe M. (2021). AAV integration in human hepatocytes. Mol. Ther..

[114] Sohlenius-Sternbeck A.-K. (2006). Determination of the hepatocellularity number for human, dog, rabbit, rat and mouse livers from protein concentration measurements. Toxicol. Vitro..

[115] Wang L., Bell P., Lin J., Calcedo R., Tarantal A.F., Wilson J.M. (2011). AAV8-mediated hepatic gene transfer in infant rhesus monkeys (Macaca mulatta). Mol. Ther..

[116] Wang L., Wang H., Bell P., McMenamin D., Wilson J.M. (2012). Hepatic gene transfer in neonatal mice by adeno-associated virus serotype 8 vector. Hum. Gene Ther..

[117] Carlson D.F., Fahrenkrug S.C., Hackett P.B. (2012). Targeting DNA With Fingers and TALENs. Mol. Ther. Nucleic Acids.

[118] Wang J.Y., Doudna J.A. (2023). CRISPR technology: A decade of genome editing is only the beginning. Science.

[119] CASGEVY® (exagamglogene autotemcel) | Patient Website https://www.casgevy.com/.

[120] Locatelli F., Lang P., Wall D., Meisel R., Corbacioglu S., Li A.M., de la Fuente J., Shah A.J., Carpenter B., Kwiatkowski J.L. (2024). Exagamglogene Autotemcel for Transfusion-Dependent β-Thalassemia. N. Engl. J. Med..

[121] Frangoul H., Locatelli F., Sharma A., Bhatia M., Mapara M., Molinari L., Wall D., Liem R.I., Telfer P., Shah A.J. (2024). Exagamglogene Autotemcel for Severe Sickle Cell Disease. N. Engl. J. Med..

[122] Simoni C., Barbon E., Muro A.F., Cantore A. (2024). In vivo liver targeted genome editing as therapeutic approach: progresses and challenges. Front. Genome.

[123] Hanlon K.S., Kleinstiver B.P., Garcia S.P., Zaborowski M.P., Volak A., Spirig S.E., Muller A., Sousa A.A., Tsai S.Q., Bengtsson N.E. (2019). High levels of AAV vector integration into CRISPR-induced DNA breaks. Nat. Commun..

[124] Paunovska K., Loughrey D., Dahlman J.E. (2022). Drug delivery systems for RNA therapeutics. Nat. Rev. Genet..

[125] Finn J.D., Smith A.R., Patel M.C., Shaw L., Youniss M.R., van Heteren J., Dirstine T., Ciullo C., Lescarbeau R., Seitzer J. (2018). A Single Administration of CRISPR/Cas9 Lipid Nanoparticles Achieves Robust and Persistent *In Vivo* Genome Editing. Cell Rep..

[126] Chen K., Han H., Zhao S., Xu B., Yin B., Lawanprasert A., Trinidad M., Burgstone B.W., Murthy N., Doudna J.A. (2024). Lung and liver editing by lipid nanoparticle delivery of a stable CRISPR–Cas9 ribonucleoprotein. Nat. Biotechnol..

[127] Buchman J.T., Hudson-Smith N.V., Landy K.M., Haynes C.L. (2019). Understanding Nanoparticle Toxicity Mechanisms To Inform Redesign Strategies To Reduce Environmental Impact. Acc. Chem. Res..

[128] Johansson J.M., Rietz H.D., Hedlund H., Eriksson H.C., Blenke E.O., Pote A., Harun S., Nordenfelt P., Lindfors L., Wittrup A. (2024). Cellular and biophysical barriers to lipid nanoparticle mediated delivery of RNA to the cytosol. bioRxiv.

[129] Cullis P.R., Felgner P.L. (2024). The 60-year evolution of lipid nanoparticles for nucleic acid delivery. Nat. Rev. Drug Discov..

[130] Polack F.P., Thomas S.J., Kitchin N., Absalon J., Gurtman A., Lockhart S., Perez J.L., Pérez Marc G., Moreira E.D., Zerbini C. (2020). Safety and Efficacy of the BNT162b2 mRNA Covid-19 Vaccine. N. Engl. J. Med..

[131] Kasiewicz L.N., Biswas S., Beach A., Ren H., Dutta C., Mazzola A.M., Rohde E., Chadwick A., Cheng C., Garcia S.P. (2023). GalNAc-Lipid nanoparticles enable non-LDLR dependent hepatic delivery of a CRISPR base editing therapy. Nat. Commun..

[132] Han X., Alameh M.-G., Xu Y., Palanki R., El-Mayta R., Dwivedi G., Swingle K.L., Xu J., Gong N., Xue L. (2024). Optimization of the activity and biodegradability of ionizable lipids for mRNA delivery via directed chemical evolution. Nat. Biomed. Eng..

[133] Lam K., Schreiner P., Leung A., Stainton P., Reid S., Yaworski E., Lutwyche P., Heyes J. (2023). Optimizing Lipid Nanoparticles for Delivery in Primates. Adv. Mater..

[134] ASGCT 27th Annual Meeting Abstracts (2024). Mol. Ther..

[135] Zakas P.M., Cunningham S.C., Doherty A., van Dijk E.B., Ibraheim R., Yu S., Mekonnen B.D., Lang B., English E.J., Sun G. (2024). *Sleeping Beauty* mRNA-LNP enables stable rAAV transgene expression in mouse and NHP hepatocytes and improves vector potency. Mol. Ther..

[136] FDA clears prime editors for testing in humans (2024). Nat. Biotechnol..

[137] Naddaf M. (2023). First trial of ‘base editing’ in humans lowers cholesterol — but raises safety concerns. Nature.

[138] Beam Therapeutics Inc (2025). A Phase 1/2 Dose-Exploration and Dose-Expansion Study to Evaluate the Safety and Efficacy of BEAM-302 in Adult Patients with Alpha-1 Antitrypsin Deficiency (AATD)-Associated Lung Disease And/or Liver Disease (clinicaltrials.Gov). https://clinicaltrials.gov/study/NCT06389877.

[139] Amabile A., Migliara A., Capasso P., Biffi M., Cittaro D., Naldini L., Lombardo A. (2016). Inheritable Silencing of Endogenous Genes by Hit-and-Run Targeted Epigenetic Editing. Cell.

[140] Cappelluti M.A., Mollica Poeta V., Valsoni S., Quarato P., Merlin S., Merelli I., Lombardo A. (2024). Durable and efficient gene silencing in vivo by hit-and-run epigenome editing. Nature.

[141] Tune Therapeutics Receives Approval to Initiate Clinical Trial in Hong Kong for HepB Epigenetic Silencer - Tune Therapeutics (2025). https://tunetx.com/tune-therapeutics-receives-approval-to-initiate-clinical-trial-in-hong-kong-for-hepb-epigenetic-silencer/.

[142] Kay M.A., Manno C.S., Ragni M.V., Larson P.J., Couto L.B., McClelland A., Glader B., Chew A.J., Tai S.J., Herzog R.W. (2000). Evidence for gene transfer and expression of factor IX in haemophilia B patients treated with an AAV vector. Nat. Genet..

[143] Buchlis G., Podsakoff G.M., Radu A., Hawk S.M., Flake A.W., Mingozzi F., High K.A. (2012). Factor IX expression in skeletal muscle of a severe hemophilia B patient 10 years after AAV-mediated gene transfer. Blood.

[144] Mingozzi F., Liu Y.-L., Dobrzynski E., Kaufhold A., Liu J.H., Wang Y., Arruda V.R., High K.A., Herzog R.W. (2003). Induction of immune tolerance to coagulation factor IX antigen by in vivo hepatic gene transfer. J. Clin. Invest..

[145] Sack B.K., Herzog R.W. (2009). Evading the immune response upon in vivo gene therapy with viral vectors. Curr. Opin. Mol. Ther..

[146] Kumar S.R.P., Biswas M., Cao D., Arisa S., Muñoz-Melero M., Lam A.K., Piñeros A.R., Kapur R., Kaisho T., Kaufman R.J. (2024). TLR9-independent CD8+ T cell responses in hepatic AAV gene transfer through IL-1R1-MyD88 signaling. Mol. Ther..

[147] Manno C.S., Pierce G.F., Arruda V.R., Glader B., Ragni M., Rasko J.J., Ozelo M.C., Hoots K., Blatt P., Konkle B. (2006). Successful transduction of liver in hemophilia by AAV-Factor IX and limitations imposed by the host immune response. Nat. Med..

[148] Mingozzi F., Maus M.V., Hui D.J., Sabatino D.E., Murphy S.L., Rasko J.E.J., Ragni M.V., Manno C.S., Sommer J., Jiang H. (2007). CD8(+) T-cell responses to adeno-associated virus capsid in humans. Nat. Med..

[149] Nathwani A.C., Tuddenham E.G.D., Rangarajan S., Rosales C., McIntosh J., Linch D.C., Chowdary P., Riddell A., Pie A.J., Harrington C. (2011). Adenovirus-associated virus vector-mediated gene transfer in hemophilia B. N. Engl. J. Med..

[150] Nathwani A.C., Gray J.T., Ng C.Y.C., Zhou J., Spence Y., Waddington S.N., Tuddenham E.G.D., Kemball-Cook G., McIntosh J., Boon-Spijker M. (2006). Self-complementary adeno-associated virus vectors containing a novel liver-specific human factor IX expression cassette enable highly efficient transduction of murine and nonhuman primate liver. Blood.

[151] Nathwani A.C., Reiss U.M., Tuddenham E.G.D., Rosales C., Chowdary P., McIntosh J., Della Peruta M., Lheriteau E., Patel N., Raj D. (2014). Long-term safety and efficacy of factor IX gene therapy in hemophilia B. N. Engl. J. Med..

[152] Dasgupta I., Keeler A.M. (2022). Rational Use of Immunosuppressive Corticosteroids in Liver-Directed Adeno-Associated Virus Gene Therapy Studies. Hum. Gene Ther..

[153] HEMGENIX® (etranacogene dezaparvovec-drlb) | Official Patient Website HEMGENIX® Etranacogene Dezaparvovec-Drlb off. Patient Website. https://www.hemgenix.com.

[154] Home | Beqvez https://beqvez.pfizerpro.com.

[155] Simioni P., Tormene D., Tognin G., Gavasso S., Bulato C., Iacobelli N.P., Finn J.D., Spiezia L., Radu C., Arruda V.R. (2009). X-Linked Thrombophilia with a Mutant Factor IX (Factor IX Padua). N. Engl. J. Med..

[156] Pipe S.W., Leebeek F.W.G., Recht M., Key N.S., Castaman G., Miesbach W., Lattimore S., Peerlinck K., Van Der Valk P., Coppens M. (2023). Gene Therapy with Etranacogene Dezaparvovec for Hemophilia B. N. Engl. J. Med..

[157] George L.A., Sullivan S.K., Giermasz A., Rasko J.E.J., Samelson-Jones B.J., Ducore J., Cuker A., Sullivan L.M., Majumdar S., Teitel J. (2017). Hemophilia B Gene Therapy with a High-Specific-Activity Factor IX Variant. N. Engl. J. Med..

[158] Cuker A., Kavakli K., Frenzel L., Wang J.-D., Astermark J., Cerqueira M.H., Iorio A., Katsarou-Fasouli O., Klamroth R., Shapiro A.D. (2024). Gene Therapy with Fidanacogene Elaparvovec in Adults with Hemophilia B. N. Engl. J. Med..

[159] ROCTAVIAN® (valoctocogene roxaparvovec-rvox) Gene Therapy Treatment for Hemophilia BioMarin Roctavian Patient EN-US. https://www.roctavian.com/en-us/.

[160] Ozelo M.C., Mahlangu J., Pasi K.J., Giermasz A., Leavitt A.D., Laffan M., Symington E., Quon D.V., Wang J.-D., Peerlinck K. (2022). Valoctocogene Roxaparvovec Gene Therapy for Hemophilia A. N. Engl. J. Med..

[161] Madan B., Ozelo M.C., Raheja P., Symington E., Quon D.V., Leavitt A.D., Pipe S.W., Lowe G., Kenet G., Reding M.T. (2024). Three-year outcomes of valoctocogene roxaparvovec gene therapy for hemophilia A. J. Thromb. Haemost..

[162] BioMarin Presents New Phase 3, Four-Year Data Underscoring Long-Term Safety and Efficacy of ROCTAVIAN® (Valoctocogene Roxaparvovec-Rvox) at International Society on Thrombosis and Haemostasis 2024 Congress https://investors.biomarin.com/news/news-details/2024/BioMarin-Presents-New-Phase-3-Four-Year-Data-Underscoring-Long-Term-Safety-and-Efficacy-of-ROCTAVIAN-valoctocogene-roxaparvovec-rvox-at-International-Society-on-Thrombosis-and-Haemostasis-2024-Congress/default.aspx.

[163] George L.A., Monahan P.E., Eyster M.E., Sullivan S.K., Ragni M.V., Croteau S.E., Rasko J.E.J., Recht M., Samelson-Jones B.J., MacDougall A. (2021). Multiyear Factor VIII Expression after AAV Gene Transfer for Hemophilia A. N. Engl. J. Med..

[164] Fong S., Yates B., Sihn C.-R., Mattis A.N., Mitchell N., Liu S., Russell C.B., Kim B., Lawal A., Rangarajan S. (2022). Interindividual variability in transgene mRNA and protein production following adeno-associated virus gene therapy for hemophilia A. Nat. Med..

[165] Sandberg H., Almstedt A., Brandt J., Castro V.M., Gray E., Holmquist L., Lewin M., Oswaldsson U., Mikaelsson M., Jankowski M.A. (2001). Structural and functional characterization of B-domain deleted recombinant factor VIII. Semin. Hematol..

[166] Siner J.I., Iacobelli N.P., Sabatino D.E., Ivanciu L., Zhou S., Poncz M., Camire R.M., Arruda V.R. (2013). Minimal modification in the factor VIII B-domain sequence ameliorates the murine hemophilia A phenotype. Blood.

[167] Factor VIII Exhibits Chaperone-dependent and Glucose-Regulated Reversible Amyloid Formation in the Endoplasmic Reticulum | Blood | American Society of Hematology https://ashpublications.org/blood/article/135/21/1899/452614/Factor-VIII-exhibits-chaperone-dependent-and. 10.1182/blood.2019002867 PMC7243144 32128578

[168] Jaffe E.A., Nachman R.L. (1975). Subunit structure of factor VIII antigen synthesized by cultured human endothelial cells. J. Clin. Invest..

[169] Hordeaux J., Lamontagne R.J., Song C., Buchlis G., Dyer C., Buza E.L., Ramezani A., Wielechowski E., Greig J.A., Chichester J.A. (2024). High-dose systemic adeno-associated virus vector administration causes liver and sinusoidal endothelial cell injury. Mol. Ther..

[170] Chand D., Mohr F., McMillan H., Tukov F.F., Montgomery K., Kleyn A., Sun R., Tauscher-Wisniewski S., Kaufmann P., Kullak-Ublick G. (2021). Hepatotoxicity following administration of onasemnogene abeparvovec (AVXS-101) for the treatment of spinal muscular atrophy. J. Hepatol..

[171] Laforet G.A. (2025). Thrombotic Microangiopathy Associated with Systemic Adeno-Associated Virus Gene Transfer: Review of Reported Cases. Hum. Gene Ther..

[172] Salabarria S.M., Corti M., Coleman K.E., Wichman M.B., Berthy J.A., D’Souza P., Tifft C.J., Herzog R.W., Elder M.E., Shoemaker L.R. (2024). Thrombotic microangiopathy following systemic AAV administration is dependent on anti-capsid antibodies. J. Clin. Invest..

[173] Zakas P.M., Brown H.C., Knight K., Meeks S.L., Spencer H.T., Gaucher E.A., Doering C.B. (2017). Enhancing the pharmaceutical properties of protein drugs by ancestral sequence reconstruction. Nat. Biotechnol..

[174] Srivastava A., Abraham A., Aboobacker F., Singh G., Geevar T., Kulkarni U., Selvarajan S., Korula A., Dave R.G., Shankar M. (2025). Lentiviral Gene Therapy with CD34+ Hematopoietic Cells for Hemophilia A. N. Engl. J. Med. 0.

[175] Wilhelm A.R., Parsons N.A., Samelson-Jones B.J., Davidson R.J., Esmon C.T., Camire R.M., George L.A. (2021). Activated protein C has a regulatory role in factor VIII function. Blood.

[176] Sternberg A.R., Martos-Rus C., Davidson R.J., Liu X., George L.A. (2024). Pre-clinical evaluation of an enhanced-function factor VIII variant for durable hemophilia A gene therapy in male mice. Nat. Commun..

[177] Brown H.C., Wright J.F., Zhou S., Lytle A.M., Shields J.E., Spencer H.T., Doering C.B. (2014). Bioengineered coagulation factor VIII enables long-term correction of murine hemophilia A following liver-directed adeno-associated viral vector delivery. Mol. Ther. Methods Clin. Dev..

[178] Unzu C., Sampedro A., Mauleón I., Alegre M., Beattie S.G., de Salamanca R.E., Snapper J., Twisk J., Petry H., González-Aseguinolaza G. (2011). Sustained Enzymatic Correction by rAAV-Mediated Liver Gene Therapy Protects Against Induced Motor Neuropathy in Acute Porphyria Mice. Mol. Ther..

[179] Sardh E., Barbaro M., Adam M.P., Feldman J., Mirzaa G.M., Pagon R.A., Wallace S.E., Amemiya A. (1993). GeneReviews®.

[180] D’Avola D., López-Franco E., Sangro B., Pañeda A., Grossios N., Gil-Farina I., Benito A., Twisk J., Paz M., Ruiz J. (2016). Phase I open label liver-directed gene therapy clinical trial for acute intermittent porphyria. J. Hepatol..

[181] Valayannopoulos V., Nicely H., Harmatz P., Turbeville S. (2010). Mucopolysaccharidosis VI. Orphanet J. Rare Dis..

[182] Ferla R., Alliegro M., Marteau J.-B., Dell’Anno M., Nusco E., Pouillot S., Galimberti S., Valsecchi M.G., Zuliani V., Auricchio A. (2017). Non-clinical Safety and Efficacy of an AAV2/8 Vector Administered Intravenously for Treatment of Mucopolysaccharidosis Type VI. Mol. Ther. Methods Clin. Dev..

[183] Brunetti-Pierri N., Ferla R., Ginocchio V.M., Rossi A., Fecarotta S., Romano R., Parenti G., Yildiz Y., Zancan S., Pecorella V. (2022). Liver-Directed Adeno-Associated Virus–Mediated Gene Therapy for Mucopolysaccharidosis Type VI. NEJM Evid..

[184] Rossi A., Romano R., Fecarotta S., Dell’Anno M., Pecorella V., Passeggio R., Zancan S., Parenti G., Santamaria F., Borgia F. (2024). Multi-year enzyme expression in patients with mucopolysaccharidosis type VI after liver-directed gene therapy. Med.

[185] Ronzitti G., Bortolussi G., van Dijk R., Collaud F., Charles S., Leborgne C., Vidal P., Martin S., Gjata B., Sola M.S. (2016). A translationally optimized AAV-UGT1A1 vector drives safe and long-lasting correction of Crigler-Najjar syndrome. Mol. Ther. Methods Clin. Dev..

[186] Dhawan A., Lawlor M.W., Mazariegos G.V., McKiernan P., Squires J.E., Strauss K.A., Gupta D., James E., Prasad S. (2020). Disease burden of Crigler-Najjar syndrome: Systematic review and future perspectives. J. Gastroenterol. Hepatol..

[187] D’Antiga L., Beuers U., Ronzitti G., Brunetti-Pierri N., Baumann U., Di Giorgio A., Aronson S., Hubert A., Romano R., Junge N. (2023). Gene Therapy in Patients with the Crigler–Najjar Syndrome. N. Engl. J. Med..

[188] admin (2024). Genethon and Hansa Biopharma Announce Initiation of a Phase 2 Trial of Imlifidase as a Pre-treatment to GNT-0003 in Severe Crigler-Najjar Syndrome. Généthon. https://www.genethon.com/genethon-and-hansa-biopharma-announce-initiation-of-a-phase-2-trial-of-imlifidase-as-a-pre-treatment-to-gnt-0003-in-severe-crigler-najjar-syndrome/.

[189] Lichter-Konecki U., Caldovic L., Morizono H., Simpson K., Ah Mew N., MacLeod E., Adam M.P., Feldman J., Mirzaa G.M., Pagon R.A., Wallace S.E., Amemiya A. (1993). GeneReviews®.

[190] Wang L., Morizono H., Lin J., Bell P., Jones D., McMenamin D., Yu H., Batshaw M.L., Wilson J.M. (2012). Preclinical evaluation of a clinical candidate AAV8 vector for ornithine transcarbamylase (OTC) deficiency reveals functional enzyme from each persisting vector genome. Mol. Genet. Metab..

[191] Ultragenyx Announces Positive Longer-term Durability Data from Two Phase 1/2 Gene Therapy Studies at American Society of Gene & Cell Therapy (ASGCT) 2022 Annual Meeting—Ultragenyx Pharmaceutical Inc. https://ir.ultragenyx.com/news-releases/news-release-details/ultragenyx-announces-positive-longer-term-durability-data-two.

[192] Ultragenyx Completes Successful End-of-Phase 2 Meeting with FDA and Finalizes Phase 3 Study Design for DTX301 Ornithine Transcarbamylase (OTC) Gene Therapy Program—Ultragenyx Pharmaceutical Inc. https://ir.ultragenyx.com/news-releases/news-release-details/ultragenyx-completes-successful-end-phase-2-meeting-fda-and.

[193] Presidential Symposium and Presentation of Top Abstracts (2023). Mol. Ther..

[194] Baruteau J., Cunningham S.C., Yilmaz B.S., Perocheau D.P., Eaglestone S., Burke D., Thrasher A.J., Waddington S.N., Lisowski L., Alexander I.E., Gissen P. (2021). Safety and efficacy of an engineered hepatotropic AAV gene therapy for ornithine transcarbamylase deficiency in cynomolgus monkeys. Mol. Ther. Methods Clin. Dev..

[195] First Clinical Data on Ornithine Transcarbamylase Deficiency Program presented at the European Society of Gene & Cell Therapy 31st Annual Meeting 2024 Demonstrates Curative Potential in First Patient Treated – Bloomsbury Genetic Therapies (2024). https://bloomsburygtx.com/first-clinical-data-on-ornithine-transcarbamylase-deficiency-program-presented-at-the-european-society-of-gene-cell-therapy-31st-annual-meeting-2024-demonstrates-curative-potential-in-first-patient/.

[196] eallison (2025). iECURE Reports Complete Clinical Response in First Infant Dosed with its In Vivo Gene Editing Candidate ECUR-506 in an Ongoing Phase 1/2 Clinical Trial in Ornithine Transcarbamylase (OTC) Deficiency | iECURE. https://iecure.com/news/iecure-reports-complete-clinical-response-in-first-infant-dosed-with-its-in-vivo-gene-editing-candidate-ecur-506-in-an-ongoing-phase-1-2-clinical-trial-in-ornithine-transcarbamylase-otc-deficiency/.

[197] Gene editor may have cured infant of a deadly metabolic disorder https://www.science.org/content/article/gene-editor-may-have-cured-infant-deadly-metabolic-disorder.

[198] Froissart R., Piraud M., Boudjemline A.M., Vianey-Saban C., Petit F., Hubert-Buron A., Eberschweiler P.T., Gajdos V., Labrune P. (2011). Glucose-6-phosphatase deficiency. Orphanet J. Rare Dis..

[199] Weinstein D.A., Derks T.G., Rodriguez-Buritica D.F., Ahmad A., Couce M.-L., Mitchell J.J., Riba-Wolman R., Mount M., Sallago J.B., Ross K.M. (2025). Safety and Efficacy of DTX401, an AAV8-Mediated Liver-Directed Gene Therapy, in Adults With Glycogen Storage Disease Type I a (GSDIa). J. Inherit. Metab. Dis..

[200] Roberts E.A., Schilsky M.L. (2023). Current and Emerging Issues in Wilson’s Disease. N. Engl. J. Med..

[201] Ultragenyx Provides Update on Stage 1 Cohorts in Pivotal Phase 1/2/3 Cyprus2+ Study Evaluating UX701 Gene Therapy for the Treatment of Wilson Disease—Ultragenyx Pharmaceutical Inc. https://ir.ultragenyx.com/news-releases/news-release-details/ultragenyx-provides-update-stage-1-cohorts-pivotal-phase-123.

[202] Murillo O., Moreno D., Gazquez C., Barberia M., Cenzano I., Navarro I., Uriarte I., Sebastian V., Arruebo M., Ferrer V. (2019). Liver Expression of a MiniATP7B Gene Results in Long-Term Restoration of Copper Homeostasis in a Wilson Disease Model in Mice. Hepatol. Baltim. Md..

[203] Vivet Therapeutics presents interim data on its Phase 1/2 GATEWAY trial for the Treatment of Wilson Disease at EASL Congress 2024 - Vivet Therapeutics https://www.vivet-therapeutics.com/vivet-therapeutics-presents-interim-data-on-its-phase-1-2-gateway-trial-for-the-treatment-of-wilson-disease-at-easl-congress-2024/.

[204] Beutler E. (1991). Gaucher’s disease. N. Engl. J. Med..

[205] Hughes D.A., Ferrante F. (2023). GALILEO-1: A Phase I/II Safety and Efficacy Study of FLT201 Gene Therapy for Gaucher Disease Type 1. Future Rare Dis..

[206] Freeline Presents Positive New Data from Phase 1/2 Trial of FLT201, Its Novel Gene Therapy Candidate for Gaucher Disease, in Late-Breaking Oral Presentation at ASGCT 27th Annual Meeting Syncona. https://www.synconaltd.com/news-and-insights/news/freeline-presents-positive-new-data-from-phase-1-2-trial-of-flt201-its-novel-gene-therapy-candidate-for-gaucher-disease-in-late-breaking-oral-presentation-at-asgct-27th-annual-meeting/.

[207] Germain D.P. (2010). Fabry disease. Orphanet J. Rare Dis..

[208] Yasuda M., Huston M.W., Pagant S., Gan L., St. Martin S., Sproul S., Richards D., Ballaron S., Hettini K., Ledeboer A. (2020). AAV2/6 Gene Therapy in a Murine Model of Fabry Disease Results in Supraphysiological Enzyme Activity and Effective Substrate Reduction. Mol. Ther. Methods Clin. Dev..

[209] Therapeutics Sangamo (2024). A Phase I/II, Multicenter, Open-Label, Single-Dose, Dose-Ranging Study to Assess the Safety and Tolerability of ST-920, an AAV2/6 Human Alpha Galactosidase A Gene Therapy, in Subjects with Fabry Disease (STAAR) (clinicaltrials.Gov). https://clinicaltrials.gov/study/NCT04046224.

[210] Sangamo Therapeutics Announces Updated Preliminary Phase 1/2 Data in Fabry Disease Clinical Study Showing Continued Tolerability and Sustained Elevated α-gal A Enzyme Activity in Five Longest Treated Patients | Sangamo Therapeutics, Inc. https://investor.sangamo.com/news-releases/news-release-details/sangamo-therapeutics-announces-updated-preliminary-phase-12-0.

[211] Hopkin R.J., Ganesh J., Bernat J., Goker-Alpan O., Nicholls K., Pahl M.V., Deegan P.B., Whitley C.B., Hughes D., Cao L. (2024). Isaralgagene civaparvovec (ST-920) gene therapy in adults with Fabry disease: Updated results from an ongoing phase 1/2 study (STAAR). Mol. Genet. Metab..

[212] Sun B., Zhang H., Benjamin D.K., Brown T., Bird A., Young S.P., McVie-Wylie A., Chen Y.T., Koeberl D.D. (2006). Enhanced Efficacy of an AAV Vector Encoding Chimeric, Highly Secreted Acid ??-Glucosidase in Glycogen Storage Disease Type II. Mol. Ther..

[213] Puzzo F., Colella P., Biferi M.G., Bali D., Paulk N.K., Vidal P., Collaud F., Simon-Sola M., Charles S., Hardet R. (2017). Rescue of Pompe disease in mice by AAV-mediated liver delivery of secretable acid α-glucosidase. Sci. Transl. Med..

[214] Sorrentino N.C., D’Orsi L., Sambri I., Nusco E., Monaco C., Spampanato C., Polishchuk E., Saccone P., De Leonibus E., Ballabio A., Fraldi A. (2013). A highly secreted sulphamidase engineered to cross the blood-brain barrier corrects brain lesions of mice with mucopolysaccharidoses type IIIA. EMBO Mol. Med..

[215] van der Ploeg A.T., Reuser A.J.J. (2008). Pompe’s disease. The Lancet.

[216] Colella P. (2024). Advances in Pompe Disease Treatment: From Enzyme Replacement to Gene Therapy. Mol. Diagn. Ther..

[217] Han S.-O., Gheorghiu D., Li S., Kang H.R., Koeberl D. (2022). Minimum Effective Dose to Achieve Biochemical Correction with Adeno-Associated Virus Vector-Mediated Gene Therapy in Mice with Pompe Disease. Hum. Gene Ther..

[218] (2023). A Phase 1 Study of the Safety of AAV2/8-LSPhGAA (ACTUS-101) in Late-Onset Pompe Disease (clinicaltrials.Gov). AskBio Inc. https://clinicaltrials.gov/study/NCT03533673.

[219] Smith E.C., Hopkins S., Case L.E., Xu M., Walters C., Dearmey S., Han S.O., Spears T.G., Chichester J.A., Bossen E.H. (2023). Phase I study of liver depot gene therapy in late-onset Pompe disease. Mol. Ther..

[220] ModernaTX, Inc. (2024). A Global, Phase 1/2, Open-Label, Dose Optimization Study to Evaluate the Safety, Pharmacodynamics, and Pharmacokinetics of mRNA-3927 in Participants with Propionic Acidemia (clinicaltrials.Gov). https://clinicaltrials.gov/study/NCT04159103.

[221] Galarreta Aima C.I., Shchelochkov O.A., Jerves Serrano T., Venditti C.P., Adam M.P., Feldman J., Mirzaa G.M., Pagon R.A., Wallace S.E., Amemiya A. (1993). GeneReviews®.

[222] Koeberl D., Schulze A., Sondheimer N., Lipshutz G.S., Geberhiwot T., Li L., Saini R., Luo J., Sikirica V., Jin L. (2024). Interim analyses of a first-in-human phase 1/2 mRNA trial for propionic acidaemia. Nature.

[223] Manganelli F., Fabrizi G.M., Luigetti M., Mandich P., Mazzeo A., Pareyson D. (2022). Hereditary transthyretin amyloidosis overview. Neurol. Sci. Off. J. Ital. Neurol. Soc. Ital. Soc. Clin. Neurol. Sci..

[224] Gillmore J.D., Gane E., Taubel J., Kao J., Fontana M., Maitland M.L., Seitzer J., O’Connell D., Walsh K.R., Wood K. (2021). CRISPR-Cas9 In Vivo Gene Editing for Transthyretin Amyloidosis. N. Engl. J. Med..

[225] Busse P.J., Christiansen S.C. (2020). Hereditary Angioedema. N. Engl. J. Med..

[226] (2025). HAELO: a Phase 3, Multinational, Randomized, Double-Blind, Placebo-Controlled Study to Evaluate the Efficacy and Safety of NTLA-2002 in Participants with Hereditary Angioedema (HAE) (clinicaltrials.gov).. Intellia Therapeutics.

[227] Longhurst H.J., Lindsay K., Petersen R.S., Fijen L.M., Gurugama P., Maag D., Butler J.S., Shah M.Y., Golden A., Xu Y. (2024). CRISPR-Cas9 In Vivo Gene Editing of *KLKB1* for Hereditary Angioedema. N. Engl. J. Med..

[228] (2024). A Two-Part Open-Label Study of REGV131-Lnp1265, A CRISPR/Cas9 Based Coagulation Factor IX Gene Insertion Therapy in Participants with Hemophilia B (clinicaltrials.Gov).. Regeneron Pharmaceuticals.

[229] Beam Therapeutics Announces Positive Initial Data for BEAM-302 in the Phase 1/2 Trial in Alpha-1 Antitrypsin Deficiency (AATD), Demonstrating First Ever Clinical Genetic Correction of a Disease-causing Mutation | Beam Therapeutics https://investors.beamtx.com/news-releases/news-release-details/beam-therapeutics-announces-positive-initial-data-beam-302-phase/.

[230] Scarpa M., Lampe C., Adam M.P., Feldman J., Mirzaa G.M., Pagon R.A., Wallace S.E., Amemiya A. (1993). GeneReviews®.

[231] Harmatz P., Prada C.E., Burton B.K., Lau H., Kessler C.M., Cao L., Falaleeva M., Villegas A.G., Zeitler J., Meyer K. (2022). First-in-human in vivo genome editing via AAV-zinc-finger nucleases for mucopolysaccharidosis I/II and hemophilia B. Mol. Ther..

[232] Manoli I., Sloan J.L., Venditti C.P., Adam M.P., Feldman J., Mirzaa G.M., Pagon R.A., Wallace S.E., Amemiya A. (1993). GeneReviews®.

[233] Chandler R.J., Venditti C.P. (2019). Gene Therapy for Methylmalonic Acidemia: Past, Present, and Future. Hum. Gene Ther..

[234] Barzel A., Paulk N.K., Shi Y., Huang Y., Chu K., Zhang F., Valdmanis P.N., Spector L.P., Porteus M.H., Gaensler K.M., Kay M.A. (2015). Promoterless gene targeting without nucleases ameliorates haemophilia B in mice. Nature.

[235] Chandler R.J., Venturoni L.E., Liao J., Hubbard B.T., Schneller J.L., Hoffmann V., Gordo S., Zang S., Ko C.W., Chau N. (2021). Promoterless, nuclease-free genome editing confers a growth advantage for corrected hepatocytes in mice with methylmalonic acidemia. Hepatology.

[236] Larrey D., Delire B., Meunier L., Zahhaf A., De Martin E., Horsmans Y. (2024). Drug-induced liver injury related to gene therapy: A new challenge to be managed. Liver Int..

[237] (2020). High-dose AAV gene therapy deaths. Nat. Biotechnol..

[238] Shieh P.B., Bönnemann C.G., Müller-Felber W., Blaschek A., Dowling J.J., Kuntz N.L., Seferian A.M. (2020). Re: “Moving Forward After Two Deaths in a Gene Therapy Trial of Myotubular Myopathy” by Wilson and Flotte. Hum. Gene Ther..

[239] Angela L., Brenda W., Allison K., Meghan B., Ma K., Shushu H., Katelyn S., Rita B.A., Rebecca A., Danielle K. (2023). Death after High-Dose rAAV9 Gene Therapy in a Patient with Duchenne’s Muscular Dystrophy. N. Engl. J. Med..

[240] Philippidis A. (2022). Novartis Confirms Deaths of Two Patients Treated with Gene Therapy Zolgensma. Hum. Gene Ther..

[241] Jiang Z., Dalby P.A. (2023). Challenges in scaling up AAV-based gene therapy manufacturing. Trends Biotechnol..

[242] Viral-vector therapies at scale: Today’s challenges and future opportunities | McKinsey. https://www.mckinsey.com/industries/life-sciences/our-insights/viral-vector-therapies-at-scale-todays-challenges-and-future-opportunities.

[243] Doxzen K.W., Adair J.E., Fonseca Bazzo Y.M., Bukini D., Cornetta K., Dalal V., Guerino-Cunha R.L., Hongeng S., Jotwani G., Kityo-Mutuluuza C. (2024). The translational gap for gene therapies in low- and middle-income countries. Sci. Transl. Med..

